# Androgen Receptor-Mediated Growth Suppression of HPr-1AR and PC3-Lenti-AR Prostate Epithelial Cells

**DOI:** 10.1371/journal.pone.0138286

**Published:** 2015-09-15

**Authors:** Young-Chae Kim, Congcong Chen, Eric C. Bolton

**Affiliations:** Department of Molecular and Integrative Physiology, University of Illinois at Urbana-Champaign, Urbana, Illinois, United States of America; Northern Institute for Cancer Research, UNITED KINGDOM

## Abstract

The androgen receptor (AR) mediates the developmental, physiologic, and pathologic effects of androgens including 5α-dihydrotestosterone (DHT). However, the mechanisms whereby AR regulates growth suppression and differentiation of luminal epithelial cells in the prostate gland and proliferation of malignant versions of these cells are not well understood, though they are central to prostate development, homeostasis, and neoplasia. Here, we identify androgen-responsive genes that restrain cell cycle progression and proliferation of human prostate epithelial cell lines (HPr-1AR and PC3-Lenti-AR), and we investigate the mechanisms through which AR regulates their expression. DHT inhibited proliferation of HPr-1AR and PC3-Lenti-AR, and cell cycle analysis revealed a prolonged G_1_ interval. In the cell cycle, the G_1_/S-phase transition is initiated by the activity of cyclin D and cyclin-dependent kinase (CDK) complexes, which relieve growth suppression. In HPr-1AR, cyclin D1/2 and CDK4/6 mRNAs were androgen-repressed, whereas CDK inhibitor, CDKN1A, mRNA was androgen-induced. The regulation of these transcripts was AR-dependent, and involved multiple mechanisms. Similar AR-mediated down-regulation of CDK4/6 mRNAs and up-regulation of CDKN1A mRNA occurred in PC3-Lenti-AR. Further, CDK4/6 overexpression suppressed DHT-inhibited cell cycle progression and proliferation of HPr-1AR and PC3-Lenti-AR, whereas CDKN1A overexpression induced cell cycle arrest. We therefore propose that AR-mediated growth suppression of HPr-1AR involves cyclin D1 mRNA decay, transcriptional repression of cyclin D2 and CDK4/6, and transcriptional activation of CDKN1A, which serve to decrease CDK4/6 activity. AR-mediated inhibition of PC3-Lenti-AR proliferation occurs through a similar mechanism, albeit without down-regulation of cyclin D. Our findings provide insight into AR-mediated regulation of prostate epithelial cell proliferation.

## Introduction

Prostate cancer is the second most prevalent cancer and the sixth leading cause of cancer mortality in men [1]. Most prostate cancer cells express the androgen receptor (AR) and are dependent on AR action for growth and proliferation [2–8]. Androgen ablation, through suppression of androgen biosynthesis and/or antagonism of AR activity, initially induces apoptosis in a subset of prostate cancer cells and suppresses growth and proliferation in those that survive, which is evidenced by tumor regression and subsequent regrowth [5,9–12]. Indeed, the proliferative actions of androgen-activated AR are well known in the mature prostate, although they are unique to neoplastic cells in this exocrine gland. However, an early event that is common among prostate cancers is a transition from AR-mediated growth suppression and differentiation of luminal epithelial cells to AR-mediated growth and proliferation of malignant versions of these cells [6].

Interestingly, the antiproliferative actions of androgen-activated AR in normal prostatic epithelia have also been demonstrated *in vivo* and in several cellular contexts. In humans and rodents, the prostatic epithelium contains basal and luminal layers interspersed with rare neuroendocrine cells. Mice lacking epithelial AR in the mature prostate develop prostate tissue that is hyperproliferative and less differentiated compared to wild-type littermates [13,14]. It has long been thought that the vast majority of cells in the basal layer, including intermediate cells, do not express AR. However, AR localization in a subset of basal cells has been reported for normal and hyperplastic prostate samples, and mounting evidence indicates that intermediate cells located within the basal layer, indeed, express AR [13–16]. In addition, compelling experiments in AR knockout mice have demonstrated that AR expression in intermediate cells is necessary for growth suppression and differentiation of these cells into luminal epithelial cells [13,14]. The proliferation and survival of intermediate cells in the basal layer are also thought to be regulated by AR-dependent signaling in prostate stroma and subsequent paracrine signaling mediated by growth and survival factors, known as andromedins, although a true andromedin remains to be identified [13,14,17–24]. Eventually, the intermediate cells migrate to the luminal layer, where AR expression is abundant [25,26]. In these cells, the activation of AR by physiologic ligands such as 5α-dihydrotestosterone (DHT) is thought to activate a gene program that suppresses proliferation and induces differentiation of intermediate cells to luminal epithelial cells that carryout secretory functions [18,27,28]. However, the mechanism whereby AR restrains cell proliferation of prostate epithelial cells is not understood.

Typically, cell proliferation is tightly associated with cell cycle regulation, and the cell cycle can be modulated at checkpoints, including the G_1_/S- and G_2_/M-phase transitions [29]. The G_1_/S-phase transition is a rate-limiting step in cell cycle regulation, and it marks the initiation of DNA synthesis, which represents commitment to the division of the parental cell into two daughter cells [30]. As a central regulator of the G_1_/S-phase transition, cyclin D expression is highly regulated such that its expression peaks in G_1_ phase. Cyclin D stimulates the G_1_/S-phase transition through association with and stabilization of cyclin-dependent kinases (CDK) 4 or 6 (CDK4/6), which are inhibited by CDK inhibitors, including CDKN1A/p21 and CDKN1B/p27 [31–33]. Complexes containing cyclin D and CDK4/6 (cyclin D-CDK4/6) are known to phosphorylate retinoblastoma protein (RB). Phosphorylation of RB (p-RB) inhibits RB-E2F complex formation and thus relieves RB-mediated growth suppression and increases expression of S phase promoting genes, including cyclin A [34,35].

Androgen activation of stably expressed AR has been shown to inhibit the proliferation of HPr-1 immortalized human prostate epithelial cells, HPrE human prostate basal epithelial cells, mPrE mouse prostate basal epithelial cells, and PC-3 prostate cancer cells [13,36–41]. Androgen also inhibits the proliferation of MCF-7 breast cancer cells, which are known to express AR [42]. In a previous study, we identified androgen responsive genes (ARGs) in HPr-1AR human prostate epithelial cells, and several of these ARGs were implicated in negative regulation of cell proliferation and progression through the cell cycle [43]. Based on these previous findings, we hypothesized that androgens and AR signaling may serve a specific role in cell cycle regulation that restrains the proliferation of prostate epithelial cells. We explore this hypothesis in the present study.

Here, we identify ARGs in prostate epithelial cells that control cell cycle progression and proliferation and investigate the mechanisms through which AR regulates their expression. We have dissected androgen action on the G_1_/S-phase transition of the cell cycle in HPr-1AR human prostate epithelial cells and PC3-Lenti-AR prostate cancer cells. We found that AR-mediated down-regulation of cyclin D-CDK4/6 complexes in both cell lines decreased RB phosphorylation and inhibited the G_1_/S-phase transition in the cell cycle, reducing cell proliferation. In HPr-1AR, AR-mediated growth suppression involves down-regulation of cyclin D-CDK complexes through transcriptional repression of cyclin D2, CDK4, and CDK6 mRNAs, destabilization of cyclin D1 mRNA, and transcriptional activation of CDKN1A. In PC3-Lenti-AR, the mechanism is limited to AR-mediated transcriptional regulation of CDK4, CDK6, and CDKN1A expression. Furthermore, overexpression of CDK4 or CDK6 suppressed DHT-inhibited cell cycle progression and proliferation in HPr-1AR and PC3-Lenti-AR, whereas CDKN1A overexpression induced cell cycle arrest in these prostate cell lines. Taken together, these data support the hypothesis that AR signaling normally functions to restrain prostate epithelial cell proliferation through cell cycle regulation.

## Materials and Methods

### Cell culture, plasmids, transfection, and viral transduction

HPr-1 cells and HPr-1AR cells were grown as described [36,43] in keratinocyte serum free medium (17005–042, Invitrogen, Carlsbad, CA) without androgen supplementation. PC-3 cells and PC3-Lenti-AR cells were grown as described [39] in RPMI Medium 1640 (11835–055, Invitrogen) with 10% fetal bovine serum (FB-11, Omega Scientific, Tarzana, CA) that was treated with charcoal-dextran to deplete steroids. HEK 293FT cells were grown in Dulbecco’s Modified Eagle Medium (DMEM, 10-017-CV, Mediatech, Manassas, VA) with 10% fetal bovine serum, 1 mM MEM sodium pyruvate (25-000-CI, Mediatech) and 500 μg/mL Geneticin (G418, 10131–035, Invitrogen). All cells were grown in a humidified 5% CO_2_ atmosphere at 37°C. Cells were treated in growth media with 0–10 nM AR agonist (5α-dihydrotestosterone, DHT) or 10 μM AR antagonist (2-hydroxyflutamide, OHF) for the indicated duration. In competition experiments, cells were cotreated with 1 nM DHT and 10 μM OHF for the indicated duration. In RNA destabilization and degradation experiments, transcription was inhibited 1 hour prior to DHT or vehicle control treatment using 1 μg/ml actinomycin D (ActD, A1410, Sigma-Aldrich, St. Louis, MO) or 20 μg/ml 5,6-dichlororibofuranosylbenzimidazole (DRB, D1916, Sigma-Aldrich).

For transient overexpression of cyclin D1 and cyclin D2, control vector pBEC118 was generated by insertion of an *Eco*R I—*Bbs* I fragment, which contains multiple cloning sites and an internal ribosome entry sequence followed by the coding sequence of the enhanced green fluorescent protein (GFP), into CSII-EF-MCS (RIKEN BioResource Center). Full-length cyclin D1 and D2 protein coding regions were amplified from HPr-1AR cDNA using PCR, and they were inserted into the *Eco*R I and *Xho* I sites of pBEC118, generating pBEC125 (CCND1) and pBEC126 (CCND2). All constructs were amplified in Stbl3 *E*. *coli* (C7373-03, Invitrogen) and transfected into HPr-1AR using Novagen GeneJuice Transfection Reagent (70967–3, EMD Millipore, Billerica, MA). The pBEC118-based vectors express enhanced green fluorescent protein (GFP), which allowed for gating and analysis of transfected cells among a background of untransfected cells.

For stable overexpression of CDK4, CDK6, and CDKN1A using lentiviral transduction, control vector pBEC127 was generated by insertion of an *Eco*R I—*Pme* I fragment, which contains multiple cloning sites and an internal ribosome entry sequence followed by the coding sequence of the monomeric red fluorescent protein (RFP), into CSII-EF-MCS (RIKEN BioResource Center). Full-length CDK4, CDK6, and CDKN1A protein coding regions were amplified from HPr-1AR cDNA or OriGene cDNA clones (OriGene Technologies, Rockville, MD) using PCR, and they were inserted into the *Eco*R I and *BamH* I sites of pBEC127, generating pBEC128 (CDK4), pBEC129 (CDK6), and pBEC130 (CDKN1A). The pBEC127-based expression vectors were amplified in Stbl3 *E*. *coli* (C7373-03, Invitrogen), transfected into HEK 293FT cells for lentivirus production, and transduced into HPr-1AR and PC3-Lenti-AR cells using pseudotyped lentivirus. The pBEC127-based vectors express monomeric red fluorescent protein (RFP), which allowed for sorting, gating, and analysis of transduced cells among a background of untransduced cells.

Briefly, HEK 293FT cells at 90–95% confluence in culture medium lacking G418 on 10-cm tissue culture dishes were cotransfected with a pBEC127-based expression vector and viral packaging vectors, which express the VSV-G, REV, GAG, and POL proteins using Lipofectamine 2000 Reagent (11668–019, Invitrogen). Culture medium lacking G418 was refreshed 12 hours after transfection. After 36 hours, the culture medium containing pseudotyped lentivirus was collected, and cell debris was removed by centrifugation at 1000×g for 15 min and filtration through a Millex-HV 0.45 μm PVDF membrane (SLHV033RS, EMD Millipore). Psedotyped lentiviruses were concentrated using polyethylene glycol precipitation [44]. Virus-containing supernatant was incubated with 8.5% polyethylene glycol 6000 (81260, Sigma-Aldrich) and 0.3 M NaCl at 4°C for 4 hours. Lentivirus was collected by centrifugation at 3000×g for 30 min, resuspended in PBS, and stored at -80°C. To generate stable cell lines overexpressing CDK4, CDK6, or CDKN1A proteins and RFP control protein, HPr-1AR and PC3-Lenti-AR cells were incubated with the corresponding lentivirus concentrate and 10 μg/mL polybrene (H9268, Sigma-Aldrich) to achieve infection efficiency ≥80%. Virus-infected cells were amplified for 1–2 weeks prior to cell sorting. For stable overexpression lines, 300,000 or more RFP-positive cells were sorted and collected using a FACSAria II cell sorter (Becton Dickinson Biosciences, Franklin Lakes, NJ). Sorted cells were amplified for two additional weeks, and the expression of the RFP, CDK4, and CDK6 proteins was validated using fluorescence microscopy and immunoblot analysis.

### Cell proliferation, apoptosis, and cell cycle analysis

The relative number of viable cells in culture was determined by quantification of ATP in metabolically active cells using CellTiter-Glo Luminescent Cell Viability Assay Kit (G7571, Promega, Madison, WI). Briefly, cells were exposed to 50% CellTiter-Glo Reagent in PBS and luminescence was analyzed using a VICTOR X5 plate reader (2030–0050, PerkinElmer, Waltham, MA).

Apoptotic cell death was assessed by flow cytometry using the Annexin V/Dead Cell Apoptosis Kit with Alexa Fluor 488-conjugated annexin V and PI for Flow Cytometry (A13201, Molecular Probes, Grand Island, NY). Briefly, cells were exposed, according to the manufacturer’s instruction, to Alexa Fluor 488-conjugated annexin V protein (annexin V), which binds with high affinity to phosphatidylserine, and propidium iodide (PI) nucleic acid binding dye. Cells were analyzed using an LSR II flow cytometer (Becton Dickinson Biosciences, Franklin Lakes, NJ) with FCS Express 4 Flow Cytometry software (De Novo Software, Glendale, CA). The percentages of cells undergoing apoptosis were determined by dual color analysis, which allowed us to distinguish three subsets of cells: viable cells (annexin V-negative and PI-negative), early apoptotic cells (annexin V-positive and PI-negative), and late apoptotic or necrotic cells (annexin V-positive and PI-positive) [45].

Cell cycle distribution was determined by flow cytometry using Vibrant DyeCycle Violet (DCV) DNA stain (V35003, Molecular Probes), which has been described previously [46]. Briefly, cells were detached with trypsin and resuspended in complete media at a cell concentration of 0.5–1 x 10^6^ cells/mL. DCV DNA stain was added to a final concentration of 5–10 μM and cells were incubated at 37°C for 40 min, protected from light. Unwashed and unfixed cells were analyzed using an LSR II flow cytometer or a FACSAria II cell sorter (Becton Dickinson Biosciences). Cell cycle distributions were then computed using FCS Express 4 Flow Cytometry software. In overexpression experiments, cells overexpressing cyclin D1 or cyclin D2 also expressed GFP, whereas cells overexpressing CDK4, CDK6, or CDKN1A also expressed RFP. Hence, corresponding gates were set to detect cell cycle distribution in the GFP-positive or RFP-positive cells, respectively.

### RNA isolation, reverse transcription, and real-time quantitative polymerase chain reaction (QPCR)

Total RNA was extracted from the cells using Qiagen RNeasy Mini Kit (74106, Qiagen) with on-column DNase treatment (79254, Qiagen). Random-primed cDNA was prepared from 1 μg of total RNA using the ProtoScript First Strand cDNA kit (E6300L, New England Biolabs, Ipswich, MA) and diluted 5-fold in water. One μl of diluted cDNA was used as template for QPCR using StepOnePlus Real-Time PCR System (Applied Biosystems, Grand Island, NY). Primers were designed using Primer3 (http://frodo.wi.mit.edu) and those that efficiently amplified [47] single products of the expected size were used for QPCR. Primer pairs for QPCR amplicons to detect mRNAs were as follows: cyclin D1 forward, 5’-GGAAGTGTTGAAGGGAGGTG-3’; cyclin D1 reverse, 5’-AACGGTAGCAGCGCAATAAG’; cyclin D2 forward, 5’-TCTTCGCTTCTGGTATCT-3’; cyclin D2 reverse, 5’-CTTGTCTGAGGAATGTTGT-3’; CDK4 forward, 5’-GAGATTACTTTGCTGCCTTA-3’; CDK4 reverse, 5’-CCCTTAGTGTAGAGAAATGG-3’; CDK6 forward, 5’-TTCCGTTGATGTGCTTAG-3’; CDK6 reverse, 5’-CTGAGAGTTGTTGGTGAT-3’; CDKN1A forward, 5’-ATCTTCTGCCTTAGTCTCA-3’; CDKN1A reverse, 5’-ACTCTTAGGAACCTCTCATT-3’. Primers for detecting pre-mRNAs were as follows: cyclin D1 forward, 5’-CATCTACACCGACAACTC-3’; cyclin D1 reverse, 5’-GGAGCAGATATGTCAGAG-3’; cyclin D2 forward, 5’-GGACATCCAACCCTACAT-3’; cyclin D2 reverse, 5’-TGGAGAGGAACAGAAATAAAG-3’; CDK4 forward, 5’-CTTGCGGCCTGTGTCTATG-3’; CDK4 reverse, 5’-GGCACTGGTTCTCATTCCTG-3’; CDK6 forward, 5’-GAGAGTGCTGGTAACTCCTTCC-3’; CDK6 reverse, 5’-AACCAAAGCCGATTCCAAG-3’; CDKN1A forward, 5’-CCGGCCAGGTAACATAGTG-3’; CDKN1A reverse, 5’-CATGGGTTCTGACGGACATC-3’. Primer pairs to detect distal 3’-UTR, proximal 3’-UTR, and exon1-exon2 junction were previously reported [48]. The QPCR was achieved using the following method: a denaturation and polymerase activation step at 94°C for 1 min and then 40 cycle consisting of 94°C for 10 s, 57°C for 10 s, and 72°C for 20 s. Data were analyzed using the comparative threshold cycle (Ct) method [49] and multiple control genes, including GAPDH, TBP, RPL19, PYGO2, NUP88 and ADAM15, which are not regulated by androgen or AR. Following normalization to control gene cDNA levels, which is reflected in the ΔCt values, the relative quantification (RQ) of the fold change for each treatment compared to reference control was determined using the following equation: RQ = 2^(–ΔCt)^ / 2^(–ΔCt reference)^. The RQ mean and standard error of the mean (SEM) were plotted using log2 scale.

### Immunoblot analysis

Immunoblots were performed to determine the relative expression of AR, cyclins, CDKs, CDKN1A, and phosphorylation of RB protein at various time points after treatment with 10 nM DHT or vehicle control. Cells were washed with PBS and harvested in 200μl of RIPA buffer (10 mM Tris-HCl (pH 8.0), 150 mM NaCl, 5% glycerol, 1 mM Na_2_EDTA, 1% Triton X-100, 0.1% SDS, 0.1% sodium deoxycholate, 2.5 mM sodium pyrophosphate, 1 mM β-glycerophosphate, 1 mM NaF and protease inhibitors). Total cell lysates containing equal amounts of protein were subjected to 4–20% mini-TGX gel (456–1096, Bio-Rad, Hercules, CA) electrophoresis and then transferred to PVDF membranes. Non-specific binding was blocked by incubation of the membranes in TBST buffer (20 mM Tris-HCl (pH 7.5), 150 mM NaCl and 0.1% Tween-20) containing 5% non-fat milk. Blots were incubated with rabbit antibodies raised against AR (06–680, EMD Millipore, Billerica, MA), β-Actin (4970, Cell Signaling, Boston, MA), CCND1 (2978, Cell Signaling), CCNE2 (4132, Cell Signaling), phospho-CDK2 (Thr160, 2561, Cell Signaling), CDK2 (2546, Cell Signaling), CDK4 (12790, Cell Signaling), CDKN1A (2947, Cell Signaling), GAPDH (2118, Cell Signaling) or mouse antibodies raised against CCND2 (ab3085, Abcam, Cambridge, MA), CCNA1 (MAB7046, R&D Systems, Minneapolis, MN), CDK4 (2906, Cell Signaling), CDK6 (3136, Cell Signaling), phospho-RB (Ser780, 9307, Cell Signaling), phospho-RB (Ser807/811, 9308, Cell Signaling), or total RB (9309, Cell Signaling), followed by incubation with anti-rabbit or anti-mouse IgG, horseradish peroxidase (HRP)-linked antibody (7074 and #7076, Cell Signaling). The blots were visualized using Pierce ECL Western Blotting Substrates (32106/34080/34096, Thermo, Rockford, IL) and HyBlot CL Autoradiography Film (E3018, Denville Scientific, Metuchen, NJ). Films were scanned and Image J software was used to quantify protein expression relative to GAPDH, β-Actin, or total RB controls.

### Statistical analysis

The significance differences among different groups were determined by one-way or two-way analysis of variances (ANOVA), followed by Tukey’s honest significant difference test. To improve the validity of ANOVA, data that displayed non-normal or heteroscedastic variance were transformed using Box-Cox power transformation [50]. A significance level of 0.05 was applied during data analysis, and different significance levels have been indicated using different lowercase letters. For time course analysis, significance differences between androgen treatment and vehicle control were determined at each time point using Student’s t-test and adjusted using the Bonferroni method. For cell cycle analysis, the proportions of G_0_/G_1_-phase cells in the different treatment groups were compared to indicate the significance differences.

## Results

### Inhibition of HPr-1AR cell proliferation in response to androgen

**Fig 1:**
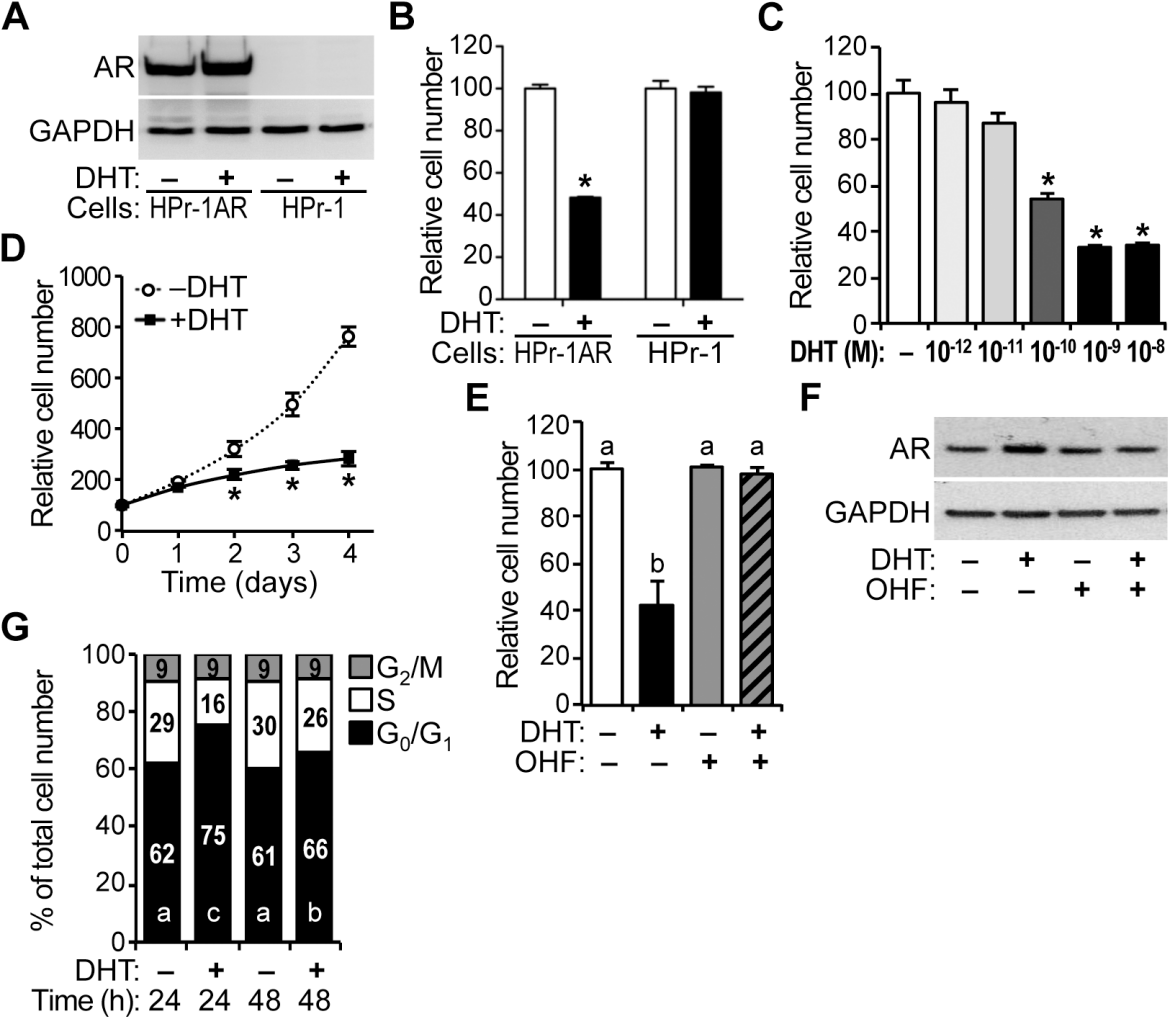
Inhibition of HPr-1AR cell proliferation in response to androgen. (A) HPr-1AR cells and parental HPr-1 cells were treated with 10 nM DHT or vehicle control for 24 hours, and immunoblots show robust AR protein expression in HPr-1AR lysates compared to HPr-1 lysates. (B) HPr-1 and HPr-1AR cells were treated with 10 nM of DHT or vehicle control, and the relative number of viable cells was determined after 72 hours of treatment by quantification of ATP in metabolically active cells. Cell number increased for all treatments, however, 52% fewer HPr-1AR cells were present following treatment with DHT compared to vehicle control. HPr-1 cell number was unaffected by DHT. Data represent the mean ± SEM, n = 4. (C) HPr-1AR cells were treated with several DHT doses (10^−12^, 10^−11^, 10^−10^, 10^−9^, 10^−8^ M) or vehicle control for 72 hours, and significant decreases in cell proliferation occurred at doses ranging from 0.1–10 nM DHT, n = 4. (D) In time course experiments, HPr-1AR cells proliferated in the presence of 10 nM DHT, albeit at a decreased rate. In comparison to control-treated HPr-1AR cells, 31%, 48%, and 63% fewer HPr-1AR cells were present following treatment with DHT for 2, 3, and 4 days (48, 72, and 96 hours), respectively. Data represent the mean ± SEM, n = 6. (E) In HPr-1AR, 2-hydroxyflutamide (OHF, 10 μM) suppressed the antiproliferative effect of 1 nM DHT at 72 hours, n = 4. (F) HPr-1AR cells were treated with various combinations of 1 nM DHT and 10 μM OHF or vehicle control for 24 hours, and immunoblots show robust AR protein expression in HPr-1AR lysates. (G) HPr-1AR cells were treated with 10 nM DHT or vehicle control, and the fraction of cells in different phases of the cell cycle was measured by DCV staining followed by FACS analysis at 24 and 48 hours (h). DHT treatment significantly increased the G_0_/G_1_ population by 13% at 24 hours and 5% at 48 hours compared to the control and decreased the S-phase and G_2_/M populations by 13% and 5%, respectively. Data represent the mean, n = 3. * *P* < 0.05. Different significance levels have been indicated using different lowercase letters.

HPr-1AR, which stably expresses wild-type AR (Fig 1A), was derived from HPr-1 human prostate epithelial cells by retroviral transduction [36,51]. Therefore, aside from the androgen sensitivity of HPr-1AR, these cell lines are isogenic. The proliferation of HPr-1AR and parental HPr-1 was compared in the presence of androgen. HPr-1AR proliferation was attenuated by 10 nM DHT, whereas HPr-1 was unaffected at 72 hours (Fig 1B). Additional experiments with HPr-1AR showed significant decreases in cell proliferation at DHT doses ranging from 0.1–10 nM (Fig 1C). In time-course experiments, HPr-1AR cells proliferated in the presence of DHT, albeit at a decreased rate in comparison to vehicle-treated cells (Fig 1D). To assess whether the decrease in cell proliferation rate was due to an increase in apoptosis, we examined cells stained with Alexa Fluor 488-annexin V, which binds phosphatidylserine, and PI nucleic acid dye following androgen treatment. DHT treatment did not significantly increase apoptosis in HPr-1AR cells (data not shown), which is consistent with the results of a previous study using mibolerone (a non-hydrolysable androgen) [36]. Also in HPr-1AR, the AR antagonist 2-hydroxyflutamide (OHF) suppressed the antiproliferative effect of DHT (Fig 1E) without decreasing AR expression (Fig 1F). Therefore, the attenuation of HPr-1AR cell proliferation in response to DHT is regulated by androgen signaling and is AR-dependent.

In addition, cell cycle distribution was quantified by Vibrant DyeCycle Violet (DCV) staining of DNA and flow cytometry analysis, which have been described previously [46]. The proportion of cells in G_0_/G_1_ phase of the cell cycle was significantly increased 13% in cells treated with DHT for 24 hours compared to control, and the S-phase population was decreased 13% leaving the G_2_/M population unchanged (Fig 1G). Similar, although more modest, effects were observed at 48 hours. Together these data indicate that the inhibition of HPr-1AR proliferation by androgen is due to a prolonged G_1_ interval in the cell cycle.

### Regulation of cyclin expression by androgen in HPr-1AR

**Fig 2:**
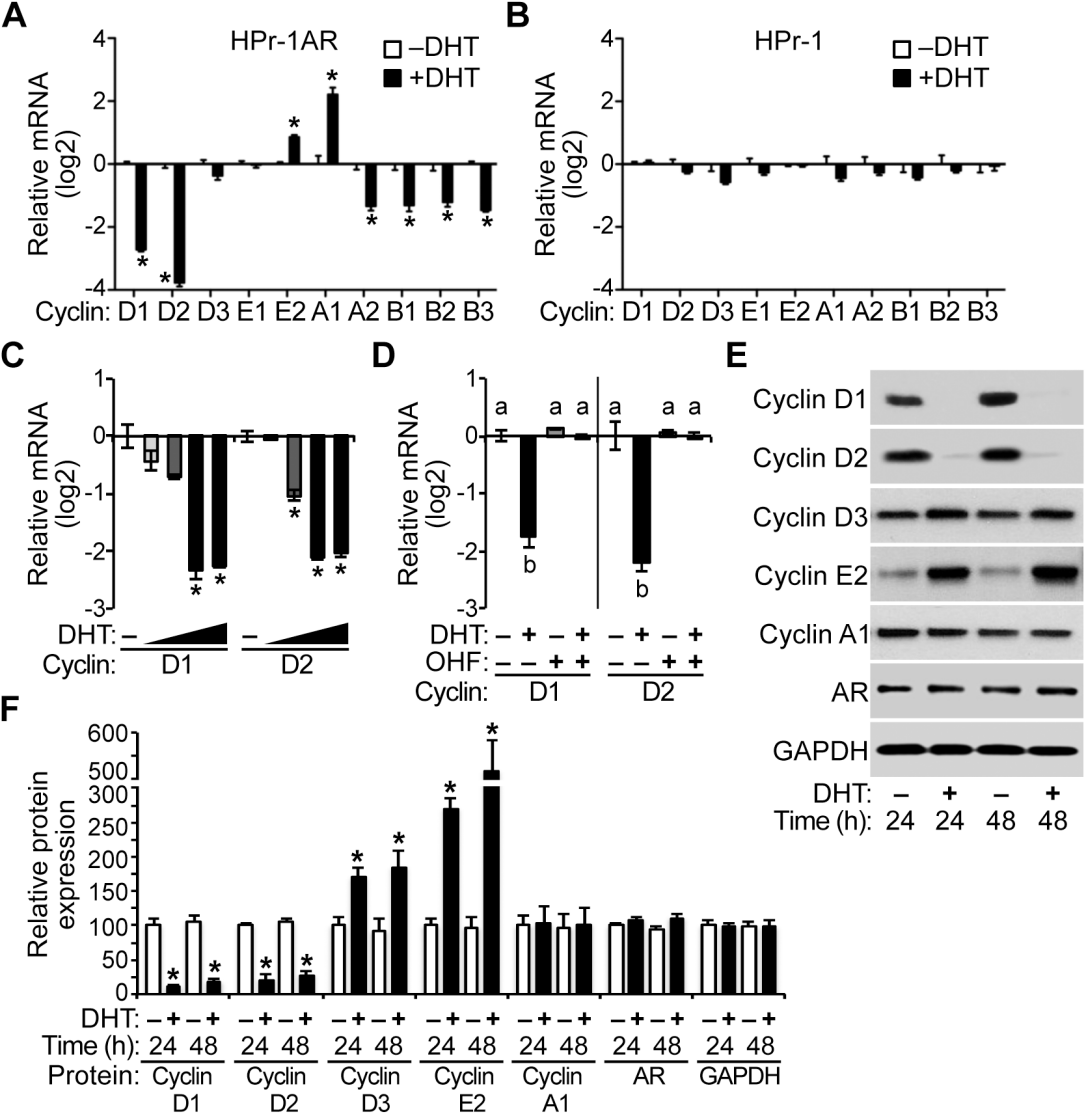
Regulation of cyclin expression by androgen in HPr-1AR. (A) After treatment with 10 nM DHT or vehicle control for 24 hours, total RNA was isolated from HPr-1AR and HPr-1 cells, cDNA was synthesized by reverse transcription and relative levels of cyclin mRNAs were quantified by QPCR analysis. Among the cyclins, cyclin D1 and D2 mRNAs were androgen-repressed 7 fold and 14 fold compared to vehicle control, respectively, and cyclin A2, B1, B2, and B3 were also androgen-repressed. In addition, cyclin E2 and A1 mRNAs were androgen-induced. Data represent the mean ± SEM, n = 3. (B) However, cyclin mRNAs were unchanged in HPr-1 cells treated with 10 nM DHT, n = 3. (C) HPr-1AR cells were treated with several DHT doses (10^−11^, 10^−10^, 10^−9^, 10^−8^ M) or vehicle control for 24 hours, and significant decreases in cyclin D1 and D2 mRNAs occurred at DHT doses ranging from 0.1–10 nM, n = 3. (D) AR antagonist 2-hydroxyflutamide (OHF, 10 μM) suppressed the down-regulation of cyclin D mRNAs by 1 nM DHT treatment at 24 hours, n = 3. (E) HPr-1AR cells were treated with 10 nM DHT or vehicle control for 24–48 hours (h), and immunoblot analysis of cell lysates reveals that cyclin D1 and D2 protein expression decreased substantially with DHT treatment compared to vehicle. (F) DHT treatment significantly decreased cyclin D1 and D2 protein levels 75% or more at 24 hours and 70% or more at 48 hours. In contrast, the expression of cyclin D3 and cyclin E2 increased 70% and 170% with DHT treatment at 24 hours, respectively, and they increased 85% and 420% at 48 hours, respectively. Relative Cyclin/GAPDH and AR/GAPDH protein ratios are shown using the mean ± SEM, n = 4. * *P* < 0.05.

Of the putative AR-regulated genes that we had previously identified in HPr-1AR [43], cyclin D1 (CCND1) was an intriguing candidate gene for the inhibition of HPr-1AR cell proliferation by androgen. However, cell cycle progression is thought to be regulated by the balance between the concentrations of activated cyclins and cyclin-dependent kinases (CDKs) [52], so we assessed the effect of androgen on the transcriptional regulation of additional cyclin and CDK genes, which were not interrogated in our previous study [43]. In HPr-1AR, the mRNA levels of several cyclin genes changed with DHT treatment (Fig 2A). Specifically, cyclin D1 and D2 mRNAs were strongly androgen-repressed, cyclin A1 was androgen-induced, and cyclins E2, A2, and B1-3 were modestly androgen-responsive. Importantly, these genes were unresponsive to androgen in the HPr-1 cells, which do not express AR (Fig 2B). Additional experiments with HPr-1AR showed significant decreases in cyclin D1 and D2 mRNAs at DHT doses ranging from 0.1–10 nM (Fig 2C), and OHF co-treatment suppressed the down-regulation of cyclin D1 and D2 mRNAs that occurred with DHT treatment (Fig 2D). A time course analysis revealed that down-regulation of cyclin D1 and D2 mRNAs occurred by 8 hours and persisted for 48 hours or more (S1 Fig). Furthermore, the down-regulation of cyclin D1 and D2 mRNAs led to decreased expression of each protein. Immunoblot analysis revealed that androgen treatment decreased cyclin D1 and D2 protein levels 75% or more at 24 hours and 70% or more at 48 hours (Fig 2E and 2F). The levels of cyclin D3 protein increased 70% or more and cyclin E2 protein increased several fold with androgen treatment by 48 hours, whereas cyclin A1 protein levels were unaffected by androgen treatment (Fig 2E and 2F).

### Attenuation of CDK expression and cyclin D-CDK4/6 activity by androgen in HPr-1AR

**Fig 3:**
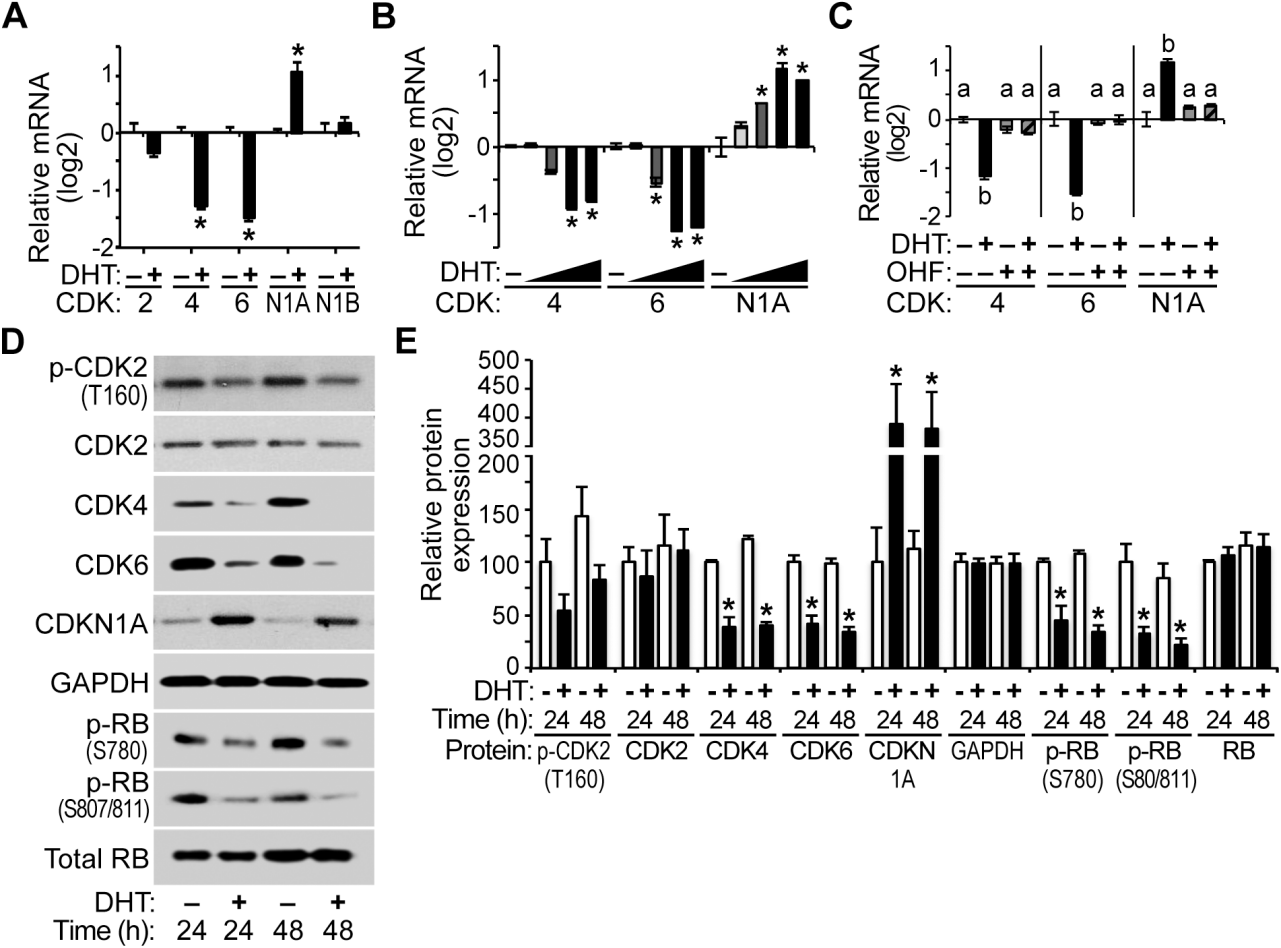
Attenuation of CDK expression and cyclin D-CDK4/6 activity by androgen in HPr-1AR. (A) HPr-1AR cells were treated with 10 nM DHT or vehicle control for 24 hours, and CDK2, CDK4 and CDK6 mRNAs and CDKN1A and CDKN1B mRNAs were quantified using QPCR. CDK4 and CDK6 mRNAs were androgen-repressed 2–3 fold. CDKN1A mRNA was DHT-induced 2-fold whereas CDKN1B mRNA was unresponsive to DHT. Data represent the mean ± SEM, n = 3. (B) HPr-1AR cells were treated with several DHT doses (10^−11^, 10^−10^, 10^−9^, 10^−8^ M) or vehicle control for 24 hours, and significant decreases in CDK4 and CDK6 mRNAs occurred at DHT doses ranging from 0.1–10 nM, n = 3. These DHT doses also increased CDKN1A mRNA. (C) AR antagonist 2-hydroxyflutamide (OHF, 10 μM) suppressed the down-regulation of CDK mRNAs and the up-regulation of CDKN1A mRNA by 1 nM DHT treatment at 24 hours, n = 3. (D) HPr-1AR cells were treated with 10 nM DHT or vehicle control for 24–48 hours (h). Immunoblot analysis of cell lysates reveals that CDK4 and CDK6 protein expression decreased and CDKN1A expression increased with DHT treatment compared to vehicle. CDK2 phosphorylation at threonine 160 (T160) decreased nearly 50% with androgen treatment at 24 and 48 hours, albeit not significantly, and total CDK2 protein expression remained unchanged. Relative to total RB expression, the levels of various phosphorylated forms of RB (S780 and S807/811) decreased over time with androgen treatment. (E) DHT treatment significantly decreased CDK4 and CDK6 protein levels 55% or more at 24 hours and 65% or more at 48 hours. The phosphorylated forms of RB declined 55% or more at 24 hours and 65% or more at 48 hours. Relative CDK/GAPDH and p-RB/total RB protein ratios are shown using the mean ± SEM, n = 4. * *P* < 0.05.

Cyclin D proteins stimulate the G_1_/S-phase transition through association with and stabilization of CDK4/6, so we examined the expression of the CDKs and CDK inhibitors that may influence the activity of cyclin D-CDK4/6 complexes. CDK 4 and 6 were androgen-repressed to a greater extent than CDK2 (Fig 3A) in HPr-1AR. For the CDK inhibitors, CDKN1A mRNA was modestly DHT-induced, and CDKN1B mRNA was unresponsive to DHT in HPr-1AR. Moreover, CDK4, CDK6, and CDKN1A mRNAs were significantly regulated at DHT doses ranging from 0.1–10 nM (Fig 3B), and OHF co-treatment suppressed the regulation of CDK4, CDK6, and CDKN1A mRNAs that occurred with DHT treatment (Fig 3C). A time course analysis revealed that the regulation of CDK4, CDK6, and CDKN1A mRNAs by androgen occurred by 16 hours and persisted for 48 hours or more (S1 Fig). Androgen treatment indeed decreased the expression of CDK4 and CDK6 proteins 55% or more at 24 hours (Fig 3D and 3E) and 65% or more at 48 hours. CDK2 protein expression was unaffected with androgen treatment, whereas CDK2 phosphorylation at threonine 160 (T160) was modestly decreased, albeit not significantly, at 24 and 48 hours. In addition, CDKN1A expression increased 280% with androgen treatment (Fig 3D and 3E). Notably, androgen has been shown to modestly increase the expression of CDKN1A and CDKN1B proteins in HPr-1AR [36], which may also contribute to the G_1_-phase arrest in HPr-1AR.

As phosphorylation of RB by cyclin D-CDK4/6 complexes initiates the G_1_/S-phase transition, we examined the relative activity of these complexes on RB phosphorylation. Our expectation was that decreased levels of cyclin D and CDK protein would lead to diminished RB phosphorylation in early G_1_ phase. In fact, RB phosphorylation at serine 780 (S780) and serine 807/811 (S807/811), which are known substrates of cyclin D-CDK4/6 complexes, was reduced 55% or more at 24 hours and 65% or more at 48 hours (Fig 3D and 3E). To further assess the effect of AR-mediated down-regulation of cyclin D1/2 and CDK4/6 proteins on the activity of cyclin D-CDK4/6, we measured the relative proliferation of HPr-1AR cells treated with androgen and PD0332991, a CDK4/6-selective kinase inhibitor, or PD0332991 by itself. Remarkably, inhibiting the kinase activity of cyclin D-CDK4/6 with PD0332991 in combination with DHT diminished cell proliferation to the same extent as DHT by itself (S2 Fig), suggesting a common biochemical pathway for PD0332991 and DHT. Indeed, the decreased expression of cyclin D1/D2 and CDK4/6 and the reduced activity of the cyclin D-CDK4/6 complexes on RB phosphorylation are correlated with the reduction in HPr-1AR proliferation, since cyclin D and CDK protein levels were reduced by androgen and cell proliferation was potently inhibited by androgen. Taken together, these data indicate that down-regulation of cyclin D-CDK4/6 complexes by AR is likely responsible for the G_1_-phase arrest of the cell cycle in HPr-1AR and attenuation of proliferation (Fig 1).

### AR-mediated destabilization of cyclin D1 mRNA in HPr-1AR

**Fig 4:**
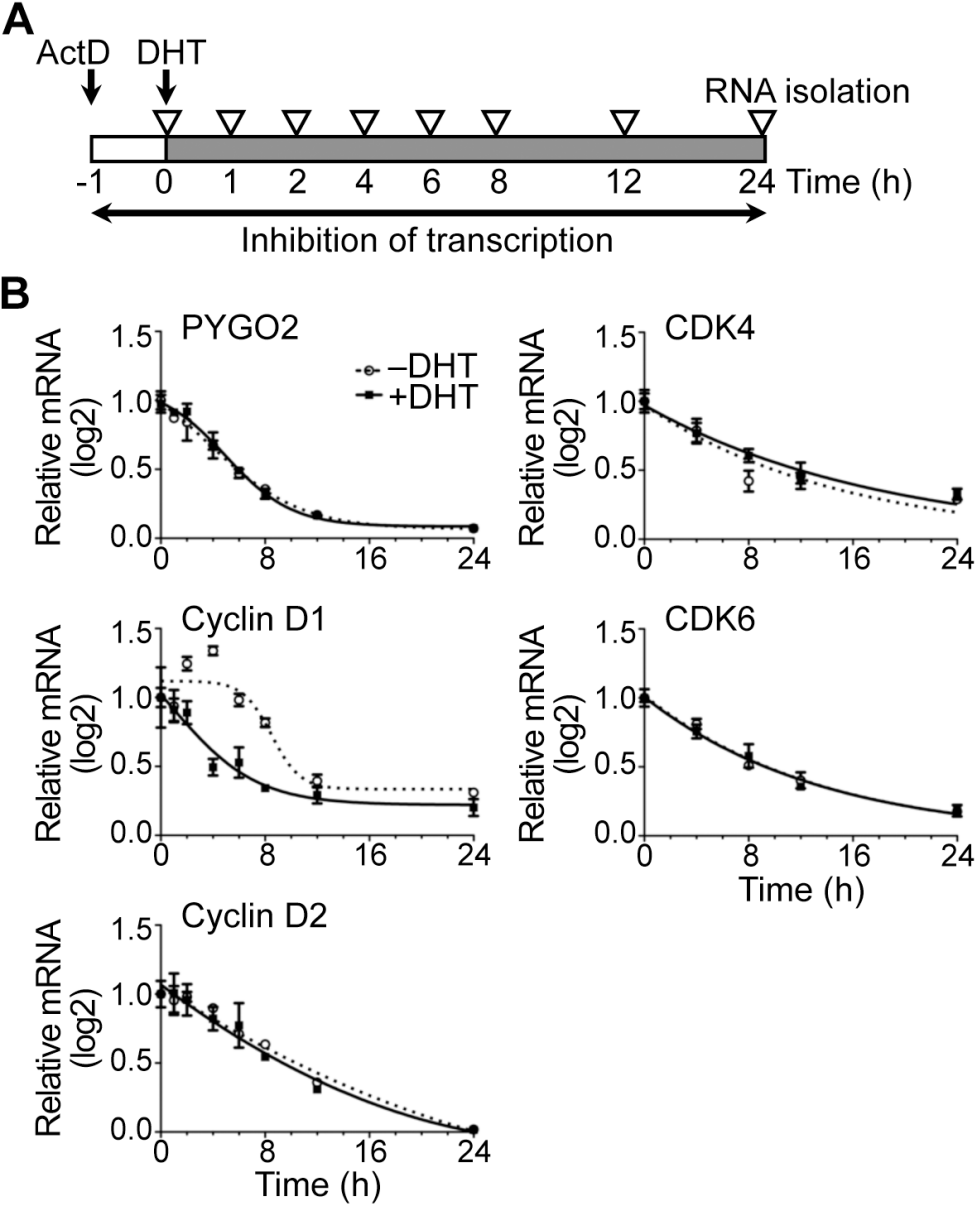
AR-mediated destabilization of cyclin D1 mRNA in HPr-1AR. (A) Experimental design scheme depicts transcriptional inhibition by actinomycin D (ActD), DHT treatment, and mRNA isolation. Cells were treated with transcription inhibitor, ActD, for 1 hour prior to treatment with 10 nM DHT or vehicle control, and total RNA was harvested at the indicated time points for quantification by QPCR. (B) Transcription of the PYGO2 control gene was unchanged by androgen, and the half-life of its mRNAs was unaffected. The half-lives of cyclin D2, CDK4, and CDK6 mRNAs were unchanged by DHT treatment compared to vehicle control, whereas the cyclin D1 mRNA half-life was 5 hours (h) in DHT-treated samples compared to 10 hours in control samples. Data represent the mean ± SEM, n = 3.

We examined several possible mechanisms for down-regulation of cyclin D1 and D2 transcripts by androgens, including direct transcriptional repression by AR at the cyclin D genes and destabilization and degradation of cyclin D transcripts. To assess whether androgen induced RNA destabilization and degradation of cyclin D and CDK mRNAs, HPr-1AR cells were treated with transcriptional inhibitor for 1 hour and then treated with 10 nM DHT or vehicle control (Fig 4A). Following transcriptional inhibition by actinomycin D (ActD), a time course of androgen or control treatment was used to determine the half-life of transcripts for the PYGO2 control gene, which was unresponsive to androgen, and transcripts for the cyclin D genes and CDK genes. Of the cyclin D mRNAs, only cyclin D1 mRNA half-life was reduced (5 hours in DHT-treated samples compared to 10 hours in control samples) with androgen treatment (Fig 4B). Similar results were observed when transcription was inhibited using 5,6-dichlororibofuranosylbenzimidazole (DRB) (S3 Fig), which inhibits transcription through a different mechanism from ActD. Thus, AR activation by DHT led to an increase in cyclin D1 mRNA decay relative to vehicle control without changing the decay rates of cyclin D2, CDK4 and CDK6 transcripts (Fig 4B and S3 Fig).

### AR-mediated transcriptional regulation of cyclin D2, CDK4/6, and CDKN1A pre-mRNAs in HPr-1AR

**Fig 5:**
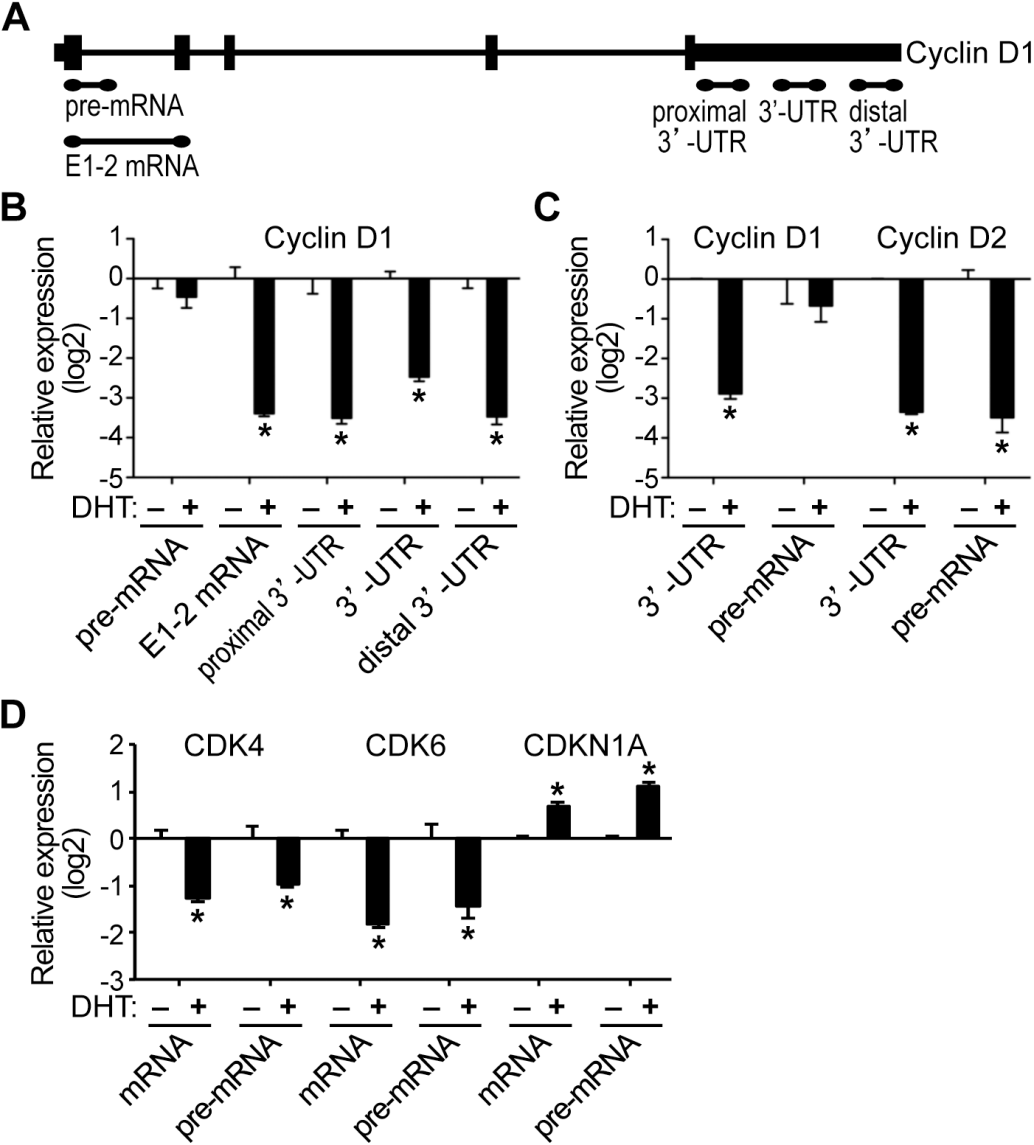
AR-mediated transcriptional regulation of cyclin D2, CDK4/6, and CDKN1A pre-mRNAs in HPr-1AR. (A) Depiction of QPCR amplicons that were designed to detect various regions of cyclin D1 pre-mRNA and mRNA. The 5’ and 3’ untranslated regions (UTRs) are depicted at each end of the cyclin pre-mRNA, vertical rectangles represent exons and horizontal lines represent introns. (B) In comparison to control samples, DHT treatment for 24 hours decreased cyclin D1 mRNAs by 6 fold or more without significant changes in cyclin D1 pre-mRNAs, indicating that AR-mediated regulation was restricted to the spliced mRNA transcripts. (C) In comparison to control samples, DHT treatment for 24 hours decreased cyclin D2 mRNA and pre-mRNA 8 fold or more, suggesting AR-mediated regulation through transcriptional repression. (D) DHT treatment for 24 hours also decreased CDK4 and CDK6 mRNAs and pre-mRNAs 2 fold or more compared to control treatment, suggesting AR-mediated regulation through transcriptional repression. In addition, CDKN1A mRNA and pre-mRNA increased in response to DHT, implicating AR-mediated regulation via transcriptional activation of CDKN1A. Data represent the mean ± SEM, n = 3. * *P* < 0.05.

In addition to the changes in mRNA levels of the cyclin D genes, we interrogated whether the down-regulation of cyclin D1 and D2 was due to transcriptional repression by AR. As precursor mRNA (pre-mRNA) is immature mRNA that has not undergone intron splicing, a change in pre-mRNA level is typically associated with transcriptional regulation rather than post-transcriptional modulation and therefore pre-mRNA serves as an indicator of ongoing transcription [53–56]. Pre-mRNA or nascent transcript levels can be used to approximate RNA polymerase transcription rates, as mRNA processing, including splicing, typically occurs within 10 minutes of the completion of transcription [57]. Pre-mRNA levels were quantified in comparison to mature RNA levels using distinct QPCR amplicons (Fig 5A–5C). For cyclin D1 transcripts, DHT treatment had little or no effect on pre-mRNA levels similar to vehicle control, whereas mature mRNAs containing sequences corresponding to E1-2 mRNA, proximal 3’-UTR, 3’-UTR, and distal 3’-UTR were all androgen-repressed (Fig 5B), indicating that full-length cyclin D1 mRNA was down-regulated in response to androgen in HPr-1AR. Alternative splicing of the cyclin D1 transcript has been described [58,59]. The cyclin D1b oncoprotein arises from alternative splicing of the CCND1 transcript, and it possesses enhanced oncogenic functions not shared by full-length cyclin D1 (cyclin D1a) [60–62]. The E1-2 mRNA amplicon was designed to detect both cyclin D1a and cyclin D1b mRNAs, whereas the 3’-UTR amplicons were designed to detect cyclin D1a mRNA. Full-length cyclin D1a mRNA was down-regulated by androgen signaling, though we cannot eliminate the possibility of cyclin D1b regulation. In striking contrast, cyclin D2 pre-mRNA was strongly DHT-repressed relative to vehicle control (Fig 5C), suggesting that the cyclin D2 gene was transcriptionally repressed by AR. Additional time course experiments, confirmed the transcriptional repression of nascent cyclin D2 transcripts upon DHT treatment, whereas cyclin D1 pre-mRNA levels were not significantly changed (S4 Fig). CDK4 and CDK6 pre-mRNAs were also DHT-repressed relative to vehicle control (Fig 5D), which suggests that AR transcriptionally represses these CDK genes. In addition, CDKN1A pre-mRNA increased in response to DHT, indicating AR-mediated transcriptional activation (Fig 5D). Taken together, the RNA stability data and the pre-mRNA data suggest that multiple different mechanisms are responsible for the down-regulation of cyclin D and CDK transcripts and up-regulation of CDKN1A mRNA in HPr-1AR. We propose that cyclin D1 mRNAs are down-regulated through a mechanism involving destabilization and degradation of the mature RNA without transcriptional repression and that cyclin D2, CDK4, and CDK6 transcripts are regulated at the pre-mRNA level through AR-mediated transcriptional repression. The up-regulation of CDKN1A mRNA is also consistent with AR-mediated transcriptional activation, which has been reported previously in prostate cancer-derived cell lines [41,63].

### Overexpression of cyclin D-CDK complex components rescues AR-mediated growth suppression of HPr-1AR

**Fig 6:**
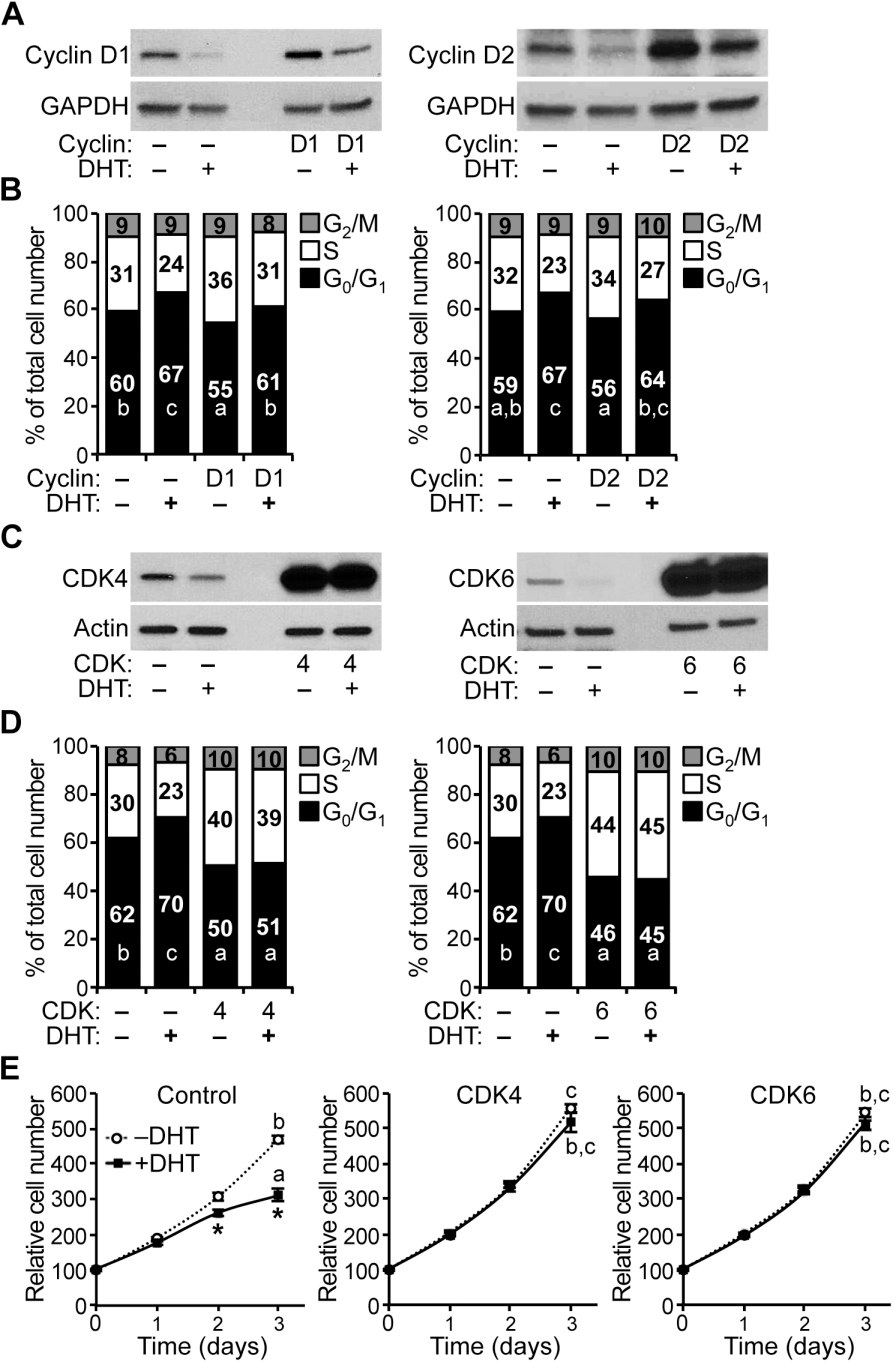
Overexpression of cyclin D-CDK complex components rescues AR-mediated growth suppression of HPr-1AR. (A) Transient overexpression of cyclin D1 and cyclin D2 was validated by immunoblot analysis. (B) Transient overexpression of either cyclin D1 or cyclin D2 decreased the proportion of HPr-1AR cells in G_0_/G_1_ phase of the cell cycle and increased the proportion of cells in S phase. However, the increases in cell cycle progression due to cyclin D1 and cyclin D2 overexpression remained completely sensitive to androgen, such that DHT treatment increased the G_0_/G_1_ populations by the same amount (6–7%) before and after transient overexpression of cyclin D1 or D2. The expression vectors used in these experiments also express green fluorescent protein, which allowed for gating and analysis of transfected cells among a background of untransfected cells. (C) Stable overexpression of CDK4 and CDK6 was validated by immunoblot analysis. (D) Stable overexpression of either CDK4 or CDK6 decreased the proportion of HPr-1AR cells in G_0_/G_1_ phase of the cell cycle and increased the proportion of cells in S phase. Moreover, the cell cycle progression after CDK4 and CDK6 overexpression became completely resistant to androgen, such that the proportions of cells in G_0_/G_1_, S, and G_2_/M phases of the cell cycle were unaffected by DHT treatment. The integrated proviral vectors used in these experiments also express red fluorescent protein, which allowed for sorting, gating and analysis of transduced cells among a background of uninfected cells. (E) Stable overexpression of either CDK4 or CDK6 increased the proliferation of HPr-1AR cells. In addition, the increases in proliferation due to CDK4 and CDK6 overexpression became completely resistant to DHT treatment. * *P* < 0.05.

To test whether changes in cyclin D expression affect the G_1_/S-phase transition of the cell cycle in HPr-1AR, we transiently overexpressed cyclin D1 or D2 and quantified the cell cycle distribution by DCV staining and flow cytometry analysis. Overexpression of cyclin D1 and D2 proteins was confirmed by immunoblot analysis (Fig 6A). For the vehicle-treated cells, overexpression of cyclin D1 or D2 decreased the proportion of cells in G_0_/G_1_ phase of the cell cycle by 5% and 3%, respectively, and increased the populations of cells in S phase and G_2_/M by 5% and 3%, respectively (Fig 6B). For the DHT-treated cells, overexpression of cyclin D1 or D2 decreased the proportion of cells in G_0_/G_1_ phase of the cell cycle by 6% and 4%, respectively, and increased the populations of cells in S phase and G_2_/M by 6% and 4%, respectively (Fig 6B). Therefore, overexpression of cyclin D1 or D2 modestly suppressed the antiproliferative effect of DHT in HPr-1AR, based on the decrease in G_0_/G_1_-phase cells and the increase in S-phase cells (Fig 6B). The AR-mediated inhibition of HPr-1AR proliferation that occurs with androgen treatment is consistent with a mechanism involving down-regulation of the expression of endogenous cyclin D1 and D2, leading to a modest decrease in the activity of cyclin D-CDK4/6 complexes in the cell cycle. Nonetheless, the modest effect of cyclin D1/2 overexpression on DHT-induced growth suppression of HPr-1AR suggests that additional AR-regulated genes are involved in this process.

To determine whether changes in CDK expression affect the G_1_/S-phase transition of the cell cycle in HPr-1AR, we stably overexpressed CDK4 or CDK6 and quantified the cell cycle distribution by DCV staining and flow cytometry analysis. Overexpression of CDK4 and CDK6 proteins was demonstrated by immunoblot analysis (Fig 6C). In comparison to vehicle-treated RFP control cells, overexpression of CDK4 or CDK6 decreased the proportion of cells in G_0_/G_1_ phase of the cell cycle by 12% and 16%, respectively, increased the populations of cells in S phase and G_2_/M by 12% and 16%, respectively (Fig 6D). In comparison to DHT-treated RFP control cells, overexpression of CDK4 or CDK6 decreased the proportion of cells in G_0_/G_1_ phase of the cell cycle by 19% and 25%, respectively, increased the populations of cells in S phase and G_2_/M by 19% and 25%, respectively (Fig 6D). Furthermore, overexpression of either CDK4 or CDK6 completely suppressed the antiproliferative effect of DHT in HPr-1AR (Fig 6E), which is consistent with the decrease in G_0_/G_1_ cells and the increase in S-phase and G_2_/M cells (Fig 6D). Therefore, we suggest the AR-mediated inhibition of HPr-1AR proliferation that occurs with androgen treatment is consistent with a mechanism involving down-regulation of the expression of endogenous CDK4 and CDK6, leading to a decrease in the activity of cyclin D-CDK4/6 complexes, delaying the G_1_/S-phase transition of the cell cycle.

In addition, we examined whether changes in CDKN1A expression affect the G_1_/S-phase transition of the cell cycle in HPr-1AR. To accomplish this, we stably overexpressed CDKN1A and assessed cell volume by quantifying forward and side light scatter using flow cytometry as described previously [64]. Overexpression of CDKN1A protein was demonstrated by immunoblot analysis (S5 Fig). In comparison to parental HPr-1AR cells and RFP control cells, which have endogenous CDKN1A expression, overexpression of CDKN1A in HPr-1AR cells increased both forward and side light scatter values (S5 Fig), indicating that these cells have increased volume relative to the control cells. Our attempts to quantify cell cycle distribution using DCV stained cells were hampered by the increased DNA content of these enlarged cells with CDKN1A overexpression (S5 Fig). Notably, HPr-1AR cells with stable CDKN1A overexpression failed to divide in culture (data not shown). Taken together, these data suggest that increased CDKN1A expression induces cell cycle arrest in HPr-1AR.

### Inhibition of PC3-Lenti-AR cell proliferation in response to androgen

**Fig 7:**
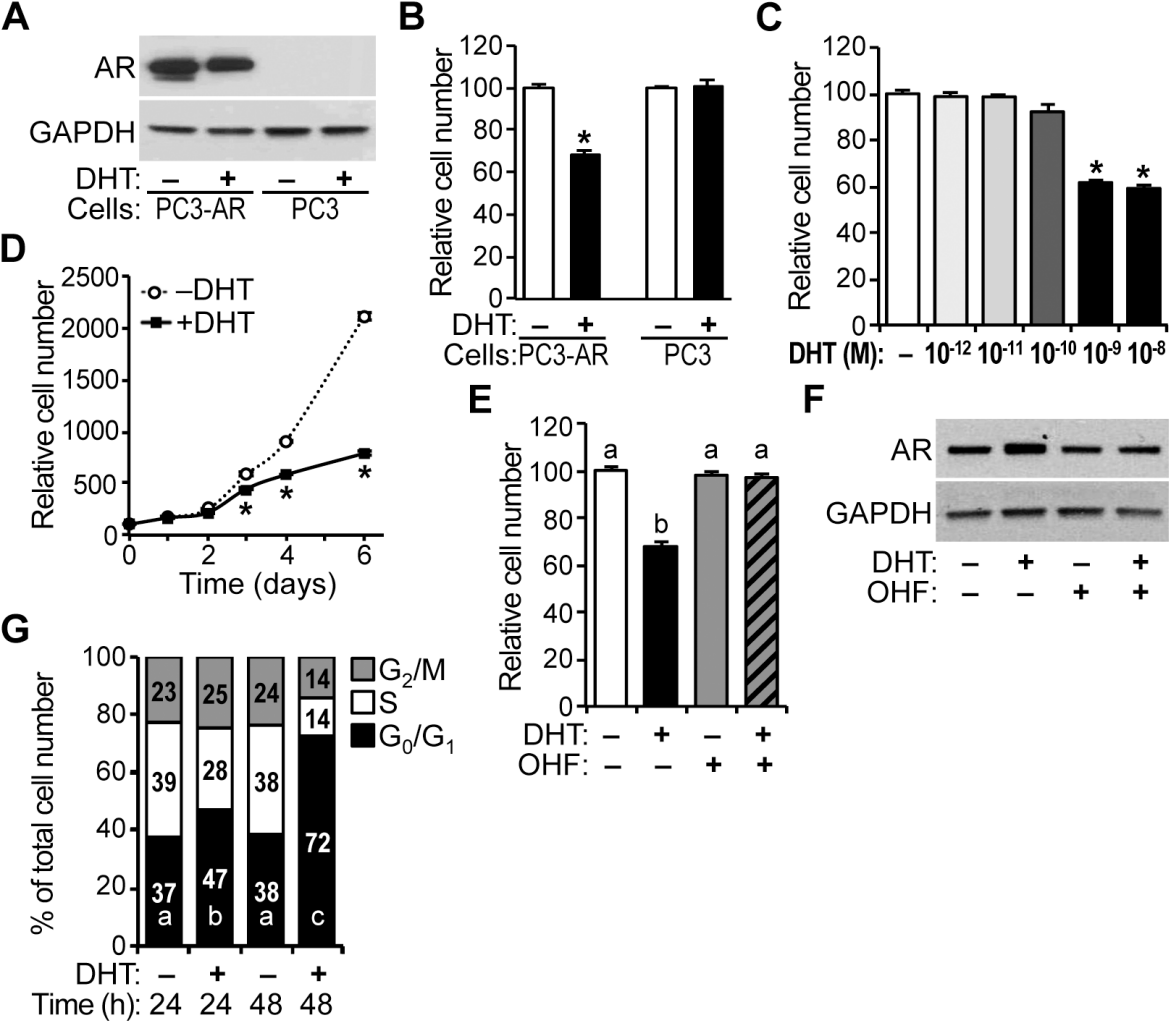
Inhibition of PC3-Lenti-AR cell proliferation in response to androgen. (A) PC3-Lenti-AR (PC3-AR) cells and parental PC-3 (PC3) cells were treated with 10 nM DHT or vehicle control for 24 hours, and immunoblots show robust AR protein expression in PC3-Lenti-AR lysates compared to PC-3 lysates. (B) PC3-Lenti-AR and PC-3 cells were treated with 10 nM of DHT or vehicle control, and the relative number of viable cells was determined after 96 hours of treatment by quantification of ATP in metabolically active cells. Cell number increased for all treatments, however, 42% fewer PC3-Lenti-AR cells were present following treatment with DHT compared to vehicle control. PC-3 cell number was unaffected by DHT. Data represent the mean ± SEM, n = 6. (C) PC3-Lenti-AR cells were treated with several DHT doses (10^−12^, 10^−11^, 10^−10^, 10^−9^, 10^−8^ M) or vehicle control for 96 hours, and significant decreases in cell proliferation occurred at doses ranging from 1–10 nM DHT, n = 6. (D) In time course experiments, PC3-Lenti-AR cells proliferated in the presence of DHT, albeit at a decreased rate. In comparison to control-treated PC3-Lenti-AR cells, 24%, 36%, and 63% fewer PC3-Lenti-AR cells were present following treatment with DHT for 3, 4, and 6 days (72, 96, and 144 hours), respectively. Data represent the mean ± SEM, n = 8. (E) In PC3-Lenti-AR, 2-hydroxyflutamide (OHF, 10 μM) suppressed the antiproliferative effect of 1 nM DHT at 96 hours, n = 6. (F) PC3-Lenti-AR cells were treated with various combinations of 1 nM DHT and 10 μM OHF or vehicle control for 24 hours, and immunoblots show robust AR protein expression in PC3-Lenti-AR lysates. (G) PC3-Lenti-AR cells were treated with 10 nM DHT or vehicle control, and the fraction of cells in different phases of the cell cycle was measured by DCV staining followed by FACS analysis at 24 and 48 hours (h). DHT treatment significantly increased the G_0_/G_1_ population by 10% at 24 hours compared to the control and decreased the S-phase population by 11%, increasing the G_2_/M population by 2%. At 48 hours, DHT treatment robustly increased the G_0_/G_1_ population by 34% compared to the control and decreased the S-phase and G_2_/M populations by 24% and 10%, respectively. Data represent the mean, n = 3. * *P* < 0.05.

For HPr-1AR, we have shown that AR regulates the expression of the cyclin D and CDK components of cyclin D-CDK4/6 complexes. As androgen activation of stably expressed AR has been shown to inhibit proliferation of PC-3 prostate cancer cells [39,41], we investigated the extent to which AR regulates cell cycle progression and cyclin D-CDK4/6 complexes in PC3-Lenti-AR. The proliferation of PC3-Lenti-AR, which stably expresses wild-type AR (Fig 7A), was inhibited by DHT treatment at 96 hours (Fig 7B), which is consistent with the findings of Litvinov et al. [39]. PC3-Lenti-AR showed significant decreases in cell proliferation at DHT doses ranging from 1–10 nM (Fig 7C). In time-course experiments, PC3-Lenti-AR cells proliferated in the presence of DHT, albeit at a decreased rate in comparison to vehicle-treated cells (Fig 7D). OHF co-treatment also suppressed the antiproliferative effect of DHT (Fig 7E) without decreasing AR expression (Fig 7F). In addition, the proportion of PC3-Lenti-AR cells in G_0_/G_1_ phase of the cell cycle was increased 10% in cells treated with DHT for 24 hours compared to control, whereas the S-phase population was decreased 11% (Fig 7G). By 48 hours, the G_0_/G_1_ population was increased 34%, and the S-phase and G_2_/M populations were decreased 24% and 14%, respectively (Fig 7G).

### Attenuation of CDK expression and cyclin D-CDK4/6 activity by androgen in PC3-Lenti-AR

**Fig 8:**
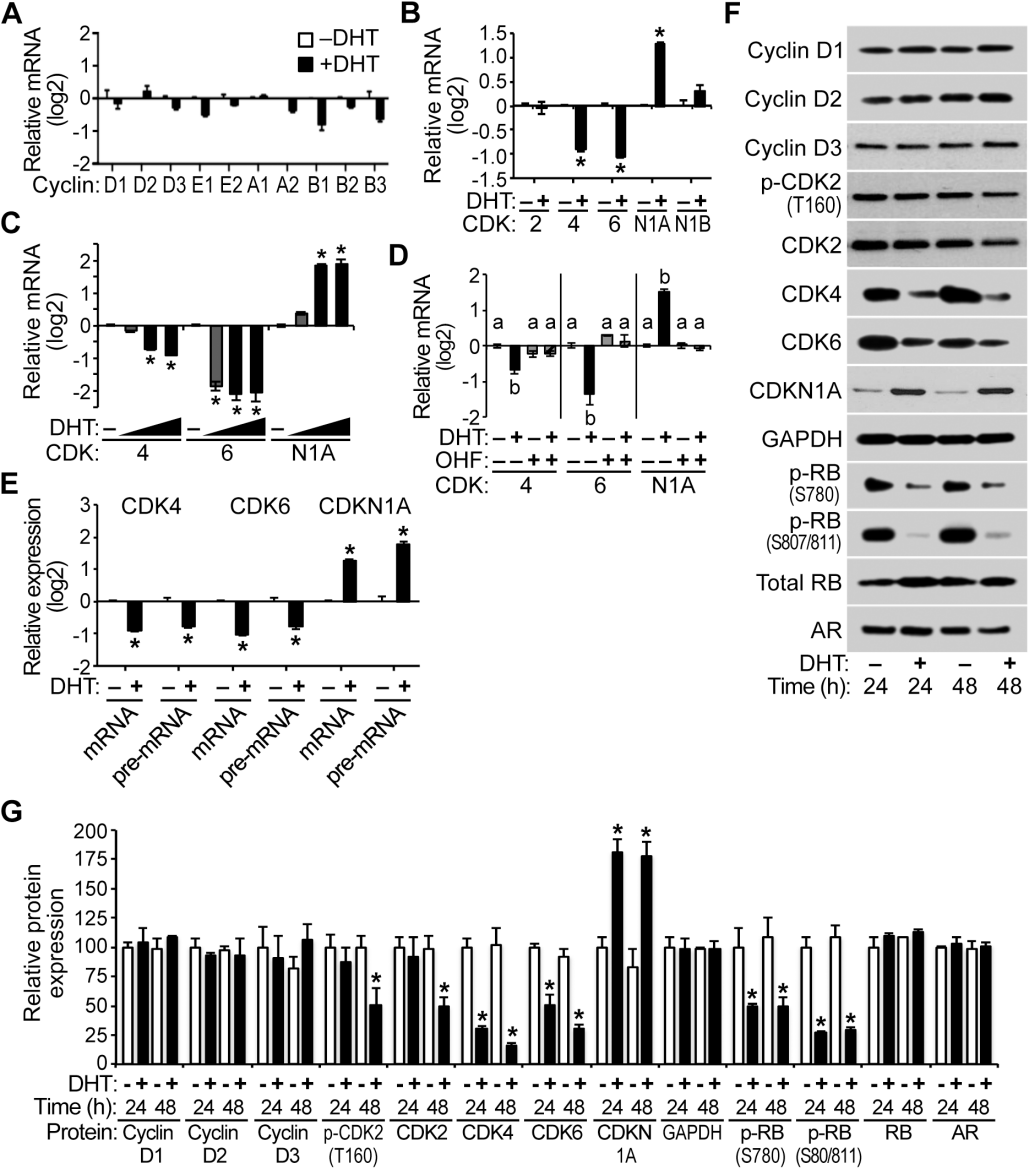
Attenuation of CDK expression and cyclin D-CDK4/6 activity by androgen in PC3-Lenti-AR. PC3-Lenti-AR cells were treated with 10 nM DHT or vehicle control for 24 hours, and (A) cyclin mRNA levels and (B) CDK and CDK inhibitor mRNA levels were determined using QPCR. The cyclin, CDK2, and CDKN1B mRNAs were unaffected by androgen treatment, whereas CDK4 and CDK6 mRNAs were androgen-repressed 1.8 fold or more. CDKN1A mRNA was DHT-induced 2.3 fold or more at 24 hours. Data represent the mean ± SEM, n = 3. (C) PC3-Lenti-AR cells were treated with several DHT doses (10^−10^, 10^−9^, 10^−8^ M) or vehicle control for 24 hours, and significant decreases in CDK4 and CDK6 mRNAs occurred at DHT doses ranging from 0.1–10 nM, n = 3. These DHT doses also increased CDKN1A mRNA. (D) AR antagonist 2-hydroxyflutamide (OHF, 10 μM) suppressed the down-regulation of CDK mRNAs and the up-regulation of CDKN1A mRNA by 1 nM DHT treatment at 24 hours, n = 3. (E) In comparison to control samples, DHT treatment for 24 hours significantly decreased CDK4 and CDK6 mRNAs and pre-mRNAs 1.5 fold or more and increased CDKN1A mRNA and pre-mRNA 2 fold or more, suggesting AR-mediated regulation of transcription at these genes, n = 3. (F) PC3-Lenti-AR cells were treated with 10 nM DHT or vehicle control for 24–48 hours (h), and immunoblot analysis of cell lysates reveals that the relative levels of the CDK proteins and various phosphorylated forms of RB (S780 and S807/811) decreased and CDKN1A expression increased over time with androgen treatment compared to vehicle. (G) CDK4 and CDK6 proteins were reduced 50% or more at 24 hours and 65% or more at 48 hours, and phosphorylated CDK2 as well as total CDK2 protein decreased 50% with androgen treatment at 48 hours. In addition, the phosphorylated forms of RB declined 50% or more at 24 hours and 55% or more at 48 hours. Relative Cyclin D/GAPDH, CDK/GAPDH and AR/GAPDH and p-RB/total RB protein ratios are shown using the mean ± SEM, n = 4. * *P* < 0.05.

In PC3-Lenti-AR, the expression of cyclin mRNAs and CDK2 and CDKN1B mRNAs were not significantly changed with DHT treatment (Fig 8A and 8B). However, CDK 4 and 6 were androgen-repressed in PC3-Lenti-AR (Fig 8B). In addition, CDKN1A mRNA was DHT-induced, which is consistent with a previous report [39]. Moreover, CDK4, CDK6, and CDKN1A mRNAs were significantly regulated at DHT doses ranging from 0.1–10 nM (Fig 8C), and OHF co-treatment suppressed the regulation of CDK4, CDK6, and CDKN1A mRNAs that occurred with DHT treatment (Fig 8D). A time course analysis revealed that the regulation of CDK4, CDK6, and CDKN1A mRNAs by androgen occurred by 8 hours and persisted for 24 hours or more (S6 Fig). CDK4 and CDK6 pre-mRNAs were also DHT-repressed relative to vehicle control (Fig 8E), implicating AR in the transcriptional repression of the CDK4 and CDK6 genes. In addition, CDKN1A pre-mRNA increased in response to DHT, implicating AR in the transcriptional activation of the CDKN1A gene (Fig 8E), which is consistent with a previous report [41].

CDK4 and CDK6 protein expression was decreased 50% or more with androgen treatment at 24 hours and 65% or more at 48 hours. Phosphorylated CDK2 and total CDK2 protein levels were also decreased 50% with DHT treatment at 48 hours (Fig 8F and 8G). Litvinov et al. reported that androgen increases CDKN1A protein expression in PC3-Lenti-AR [39]. Consistent with this report, we found that CDKN1A protein was indeed DHT-induced approximately 2 fold (Fig 8F and 8G). Furthermore, RB phosphorylation at S780 and S807/811 was decreased 50% or more at 24 hours and 55% or more at 48 hours, and total RB protein levels were unaffected by androgen (Fig 8F and 8G). Taken together, the proliferation inhibition (Fig 7B) and G_1_-phase arrest (Fig 7G) programs that are activated by androgen action and the inhibition of RB phosphorylation suggest that the AR-mediated inhibition of PC3-Lenti-AR proliferation likely involves the regulation of cyclin D-CDK4/6 complexes, delaying the G_1_/S-phase transition of the cell cycle. Hence, we propose that novel AR-mediated transcriptional repression of CDK4/6 expression together with elevated levels of CDK inhibitor CDKN1A are responsible for diminished RB phosphorylation and the AR-mediated inhibition of PC3-Lenti-AR proliferation that occurs with androgen treatment, which is consistent with the findings of Litvinov et al. and Mirochnik et al. [39,41].

### Overexpression of cyclin D-CDK complex components partially rescues AR-mediated growth suppression of PC3-Lenti-AR

**Fig 9:**
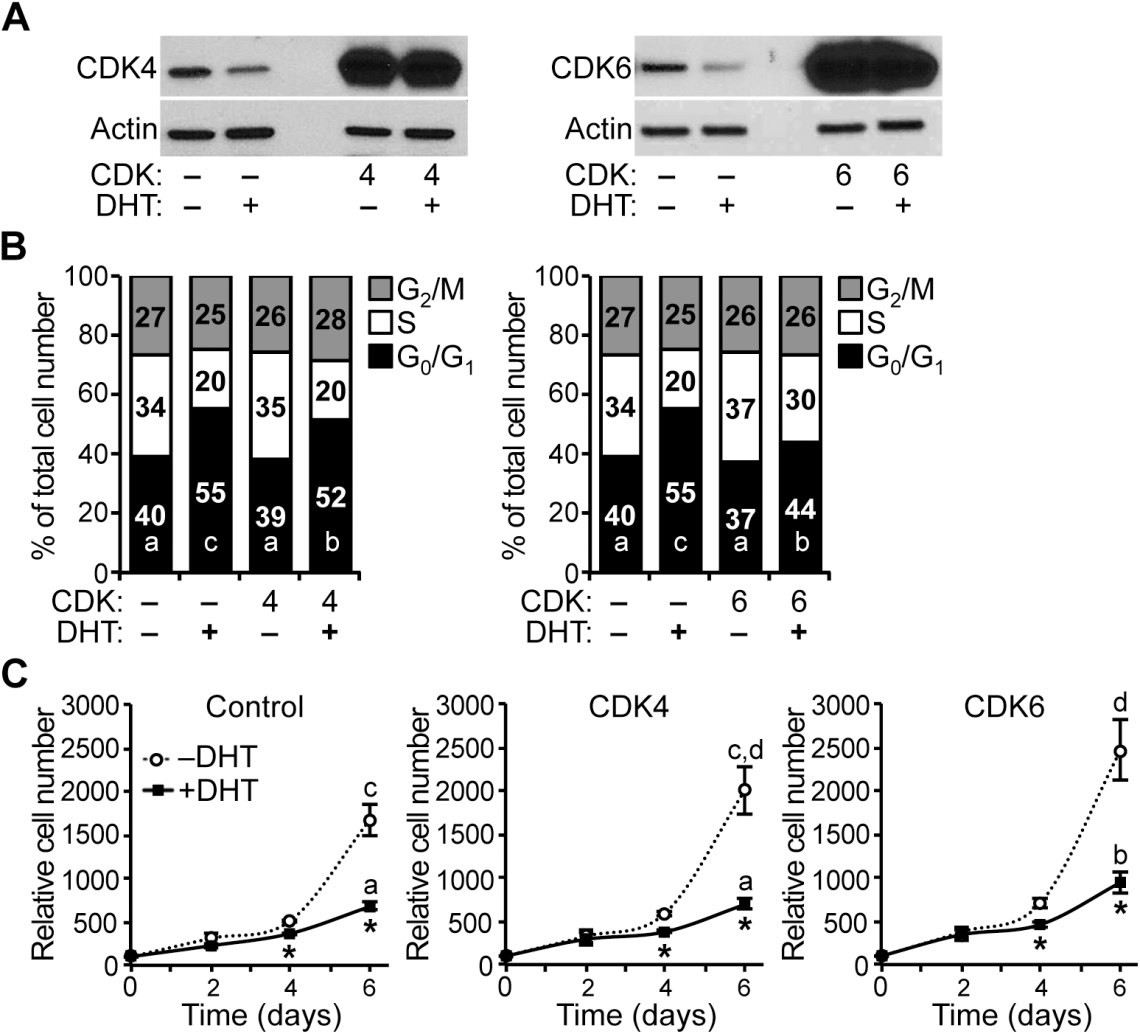
Overexpression of cyclin D-CDK complex components partially rescues AR-mediated growth suppression of PC3-Lenti-AR. (A) Stable overexpression of CDK4 and CDK6 was validated by immunoblot analysis. (B) In the absence of DHT, stable overexpression of CDK4 and CDK6 did not significantly decrease the proportion of PC3-Lenti-AR cells in G_0_/G_1_ phase of the cell cycle. However, in the presence of 10 nM DHT, overexpression of CDK4 and CDK6 partially but significantly attenuates the DHT-induced cell cycle arrest by 2% and 8%, respectively. The integrated viral vectors used in these experiments also express red fluorescent protein, which allowed for sorting, gating and analysis of transduced cells among a background of uninfected cells. (C) Stable overexpression of either CDK4 or CDK6 increased the proliferation of PC3-Lenti-AR cells. In addition, the increases in proliferation due to CDK4 and CDK6 overexpression remained partially sensitive to DHT treatment. For the relative cell numbers on treatment day 6, the significance differences among different overexpression groups (Control, CDK4, CDK6) and treatments (–DHT, +DHT) were determined by two-way ANOVA, followed by Tukey’s honest significant difference test. Box-Cox power transformation was used to stabilize variance and improve the validity of ANOVA. * *P* < 0.05.

To determine whether changes in CDK expression affect the G_1_/S-phase transition of the cell cycle in PC3-Lenti-AR, we stably overexpressed CDK4 or CDK6 and quantified the cell cycle distribution by DCV staining and flow cytometry analysis. Overexpression of CDK4 and CDK6 proteins was confirmed by immunoblot analysis (Fig 9A). In comparison to vehicle-treated RFP control cells, overexpression of CDK4 or CDK6 did not significantly change the proportion of PC3-Lenti-AR cells in the different phases of the cell cycle (Fig 9B). In comparison to DHT-treated RFP control cells, overexpression of CDK4 or CDK6 decreased the proportion of cells in G_0_/G_1_ phase of the cell cycle by 3% and 11%, respectively, increased the populations of cells in S phase and G_2_/M by 3% and 11%, respectively (Fig 9B). Furthermore, CDK6 overexpression partially suppressed the antiproliferative effect of DHT in PC3-Lenti-AR (Fig 9C), which is consistent with the decrease in G_0_/G_1_ cells and the increase in S-phase and G_2_/M cells (Fig 9B). CDK4 overexpression showed a similar trend but did not significantly suppress the antiproliferative effect of DHT in PC3-Lenti-AR (Fig 9C). Therefore, we suggest the AR-mediated inhibition of PC3-Lenti-AR proliferation that occurs with androgen treatment is consistent with a mechanism involving down-regulation of endogenous CDK4 and CDK6 expression, leading to a decrease in the activity of cyclin D-CDK4/6 complexes in the cell cycle.

We also examined whether changes in CDKN1A expression affect the G_1_/S-phase transition of the cell cycle in PC3-Lenti-AR. To do this, we stably overexpressed CDKN1A and assessed cell volume by quantifying forward and side light scatter using flow cytometry. Overexpression of CDKN1A protein was confirmed by immunoblot analysis (S7 Fig). In comparison to parental PC3-Lenti-AR cells and RFP control cells, which have endogenous CDKN1A expression, overexpression of CDKN1A in PC3-Lenti-AR cells increased both forward and side light scatter values (S7 Fig), indicating that these cells have increased volume relative to the control cell lines. Our attempts to quantify cell cycle distribution using DCV stained cells were again thwarted by the increased DNA content of the enlarged cells with CDKN1A overexpression (S7 Fig). Notably, PC3-Lenti-AR cells with stable CDKN1A overexpression failed to divide in culture (data not shown). These finding indicate that increased CDKN1A expression induces cell cycle arrest in PC3-Lenti-AR, which is consistent with a previous report by Mirochnik et al. [41].

## Discussion

**Fig 10:**
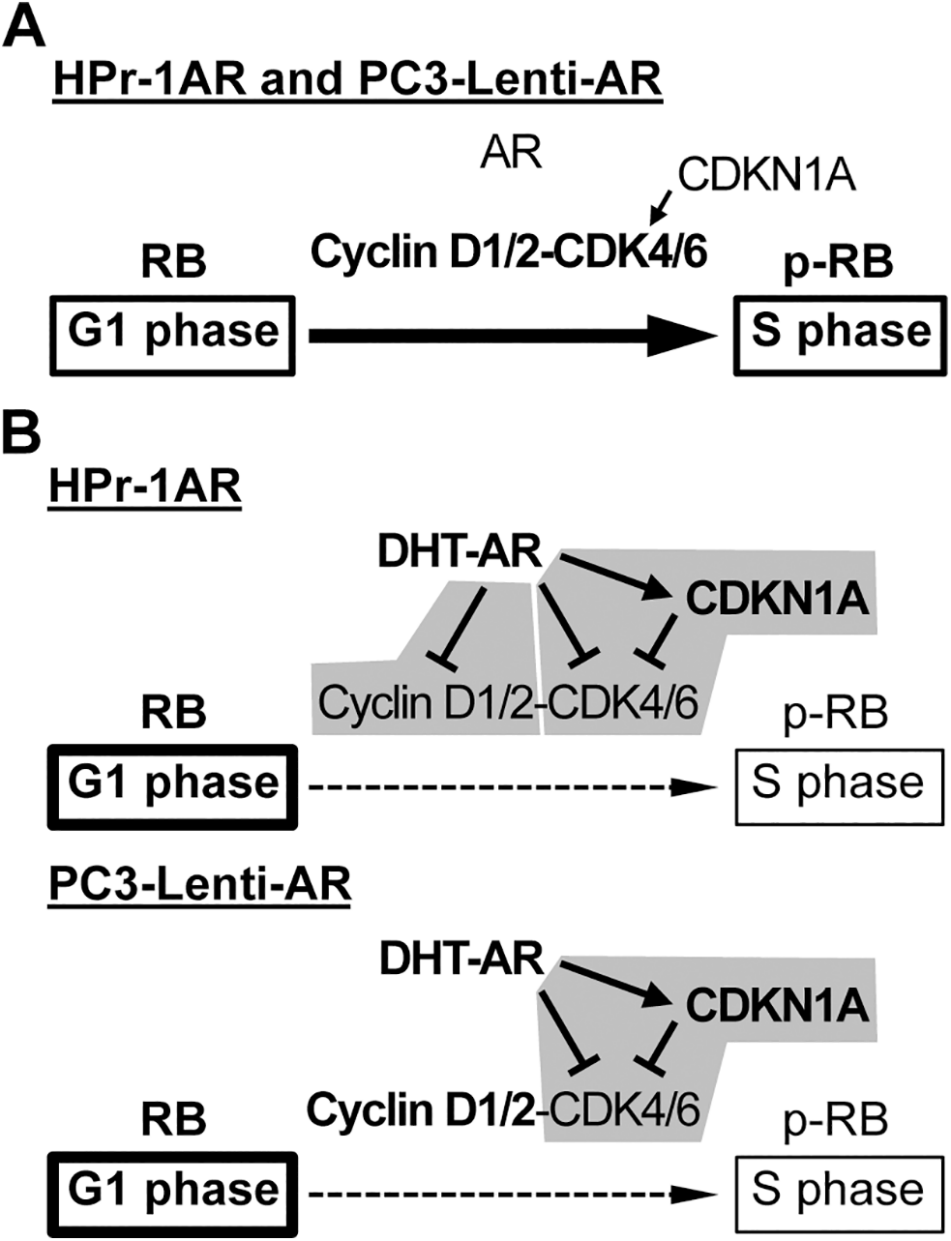
Model for AR-mediated growth suppression of HPr-1AR and PC3-Lenti-AR. (A) In the absence of androgen, cell cycle progression through the G_1_/S-phase transition and proliferation of HPr-1AR and PC3-Lenti-AR are promoted by basal expression of cyclin D1/2, CDK4/6, and CDKN1A. (B) Androgen signaling through AR increases CDKN1A expression and decreases the abundance and activity of cyclin D-CDK4/6 complexes by multiple mechanisms. Common mechanism for HPr-1AR and PC3-Lenti-AR: CDK4 and CDK6 are down-regulated through AR-mediated transcriptional repression. In addition, CDKN1A expression is up-regulated by AR-mediated transcriptional activation, and at elevated levels, CDKN1A inhibits CDK4/6-mediated phosphorylation of RB. Therefore, cell cycle progression through the G_1_/S-phase transition is delayed and proliferation is inhibited. HPr-1AR-specific mechanism: Cyclin D1 mRNA is down-regulated through an AR-mediated mRNA decay mechanism, whereas cyclin D2 pre-mRNA is down-regulated by AR-mediated transcriptional repression.

The antiproliferative actions of androgens in non-malignant prostate epithelial cells have been demonstrated *in vivo* and in several cellular contexts, although the mechanism whereby AR restrains cell proliferation of prostate epithelial cells is not understood. DHT inhibited HPr-1AR and PC3-Lenti-AR cell proliferation, and cell cycle analysis revealed a prolonged G_1_ interval in response to androgen (Figs 1 and 7). We found multiple genes involved in cell cycle progression and proliferation to be regulated by AR in HPr-1AR and PC3-Lenti-AR (Figs 2, 3 and 8). AR-dependent mechanisms decreased the expression and activity of cyclin D1/2-CDK4/6 complexes on RB phosphorylation. In HPr-1AR, cyclin D1 mRNA was destabilized and exhibited a shorter half-life following androgen treatment, whereas cyclin D2, CDK4, and CDK6 mRNAs were transcriptionally repressed (Figs 4 and 5). Similar AR-mediated down-regulation of CDK4 and CDK6 nascent transcripts occurred in PC3-Lenti-AR (Fig 8). In addition, CDKN1A pre-mRNA was androgen-induced in both prostate cell lines (Figs 3 and 8). Furthermore, overexpression of CDK4 or CDK6 suppressed DHT-inhibited cell cycle progression and proliferation in HPr-1AR and PC3-Lenti-AR (Figs 6 and 9), whereas CDKN1A overexpression induced cell cycle arrest (S5 and S7 Figs). We therefore propose that the mechanism responsible for AR-mediated inhibition of HPr-1AR and PC3-Lenti-AR cell proliferation involves down-regulation of cyclin D-CDK4/6 complexes through transcriptional repression of the CDK4 and CDK6 genes and transcriptional activation of the CDKN1A gene (Fig 10). We also propose that AR-mediated decreases in cyclin D expression via cyclin D1 mRNA decay and cyclin D2 transcriptional repression contribute to HPr-1AR growth suppression (Fig 10).

### AR-mediated regulation of prostate epithelial cell proliferation

Previous studies comparing PC3-Lenti-AR to PC-3 and HPr-1AR to HPr-1 have shown that AR expression is necessary for androgen-induced growth suppression of these cell lines [36,39]. In the present study, we have provided substantial evidence for the mechanism of AR-mediated growth suppression using these prostate cell lines. Both PC3-Lenti-AR and HPr-1AR were derived from androgen-insensitive prostate epithelial cell lines, but they are distinguished by their ability to form tumors. PC-3 cells are castration-resistant prostate adenocarcinoma cells that have high metastatic potential, whereas HPr-1 cells are immortalized, benign prostate epithelial cells [36,37,39,51,65]. In addition, stable expression of AR in PC-3 prostate cancer cells profoundly suppressed cell proliferation and tumor growth in intact-male mouse xenografts [39].

Many prostate cancer cells derived from tumor parenchyma express AR, and they are somewhat dependent on AR activation for growth and proliferation [2–8]. Androgen action and cell cycle progression has been examined in AR-immunoreactive cancer cells. Androgen ablation *in vivo* initially induces apoptosis in a subset of prostate cancer cells, leading to tumor regression, and induces G_1_-phase arrest in those that survive, which is evidenced by subsequent tumor regrowth [5,9–12]. However, prostate cancer cells derived from castrated patients, such as LNCaP, LAPC-4, MDA-PC-2B, VCAP, and so on, are dependent upon androgens for growth and proliferation, and they are thought to have mechanisms of castration resistance (including AR mutation, amplification, and constitutively active splice variants) as a strategy to survive in the presence of castrate levels of androgen [66–69]. Studies using castration-resistant LNCaP cells have reported that androgen stimulates the proliferation of prostate cancer cells by enhancing the expression of G_1_/S regulatory proteins, whereas androgen withdrawal causes G_1_-phase arrest [3,5,70–73]. Indeed, the proliferative actions of androgen-activated AR are well known in malignant epithelial cells of prostate origin. However, one should not conclude that androgen is a positive proliferative signal in all prostate epithelial cells. For example, E006AA and PC-3 prostate cancer cells display a complete absence of AR growth-regulatory function due to loss of AR-dependent growth suppression [37–40,74]. Therefore, prostate cancer cell lines like LNCaP (AR-positive) and PC-3 (AR-negative) may have evolved/adapted gene expression programs, through AR-mutation or p53-mutation, to escape the growth suppressive actions of androgens [39,75–77].

AR signaling has been suggested to function as a negative regulator of epithelial proliferation in the prostate gland. Such a role for AR is supported by compelling experiments in AR knockout mice that have demonstrated that AR expression in intermediate cells is necessary for growth suppression and differentiation of these cells into luminal epithelial cells [13,14]. Ling et al. reported that immortalized non-tumorigenic HPr-1AR cells undergo growth suppression and cytodifferentiation in response to mibolerone (a non-hydrolysable androgen analog) [36], which is consistent with our results that the endogenous androgen DHT is a potent inhibitor of their proliferation (Fig 1). Importantly, normal secretory luminal epithelial cells in the prostate, which express AR, are well-differentiated and quiescent, as androgen does not stimulate their proliferation [28,78]. Thus, AR signaling restrains proliferation of normal prostate epithelial cells.

### AR-mediated regulation of cell cycle progression

Here, we have provided evidence for AR-mediated regulation of cell cycle progression at the G_1_/S-phase transition as a mechanism for growth suppression of HPr-1AR and PC3-Lenti-AR (Figs 1–3 and 7 and 8). In the cell cycle, G_1_-phase progression is regulated by the expression, formation, and activation of cyclin-CDK complexes. Cyclin D-CDK4/6 complexes are important for the G_1_/S-phase transition because they increase RB-phosphorylation, activation of E2F transcription factors and expression of S phase promoting genes [34,35]. In addition, we discovered that DHT-induced inhibition of HPr-1AR and PC3-Lenti-AR proliferation (Figs 1 and 7) is associated with the reduced expression and activity of cyclin D1/2-CDK4/6 complexes on RB phosphorylation (Figs 3 and 8). Furthermore, stable overexpression of CDK4/6 completely suppresses the DHT-induced G_1_-phase delay and restrained proliferation of HPr-1AR cells (Fig 6) and partially suppressed the DHT-induced G_1_-phase delay and inhibited proliferation of PC3-Lenti-AR cells (Fig 9). Taken together, these results suggest that AR-mediated down-regulation of cyclin D-CDK4/6 complexes is a crucial mechanism through which androgen signaling inhibits cell cycle progression and exerts growth suppression in non-tumorigenic HPr-1AR and invasive PC3-Lenti-AR cells. The delay in cell cycle progression at the G_1_/S-phase transition and growth suppression in these cells likely involve multiple AR-mediated mechanisms that serve to regulate the expression and activity of cyclin-CDK complexes.

Of the G_1_/S-phase cyclins, the expression of cyclin D1 and cyclin D2 was substantially decreased and cyclin D3 protein was modestly increased in androgen-treated HPr-1AR (Figs 2, 4 and 5), whereas their expression was unaffected by androgen in PC3-Lenti-AR (Fig 8). In HPr-1AR, we also found that cyclin D1/2 overexpression stimulated G_1_/S-phase progression and modestly suppressed the DHT-induced G_1_-phase delay in these epithelial cells (Fig 6B), which led us to conclude that AR-mediated regulation of cyclin D1/2 expression modulates G_1_/S-phase progression and growth suppression. Additional support for this conclusion comes from the finding that cyclin D1 overexpression in LNCaP prostate cancer cells increased the fraction of S-phase cells and decreased growth factor requirements for their proliferation [71]. Cyclin D3 has been shown to positively regulate terminal cytodifferentiation in multiple tissues, including skeletal muscle and liver [79–82]. Therefore, the up-regulation of cyclin D3 may also contribute to the androgen-induced differentiation and growth suppression that was previously reported for HPr-1AR [36]. Several additional G_1_/S-phase cyclins that partner with CDK2 were androgen responsive in HPr-1AR cells. Cyclin E2 expression was DHT-induced, whereas cyclin A2 and B1-3 mRNAs were modestly DHT-repressed (Fig 2). The altered expression of these cyclins in HPr-1AR might affect CDK2 stability and activity. However, the levels of phosphorylated CDK2 and total CDK2 expression remained unchanged with androgen treatment (Fig 3), suggesting that CDK2 stability and activity were unaffected by the altered expression of cyclins A, B, and E in DHT-treated HPr-1AR cells. While we cannot eliminate the possibility that CDKN1A up-regulation suppresses CDK2 activity in HPr-1AR, our results demonstrate the importance of cyclin D-CDK4/6 complexes and the G_1_/S-phase transition in AR-mediated growth suppression.

In human non-transformed prostate epithelial cells, the expression of p53 and CDKN1A has been shown to increase with androgen treatment [83]. Consistent with previous reports [36,39], we observed CDKN1A up-regulation in DHT-treated HPr-1AR and PC3-Lenti-AR cells (Figs 3 and 8). These findings support the idea that G_1_-phase progression is further regulated by cellular signals that activate p53 and increase the expression of CDK inhibitors, including CDKN1A. At low concentrations (i.e., basal expression), CDKN1A has been shown to promote assembly of active cyclin D-CDK complexes and G_1_/S-phase transition, whereas at higher concentrations (i.e., induced expression), CDKN1A has been shown to inhibit the activity of cyclin E-CDK2 and cyclin D-CDK4/6 complexes and decrease cell proliferation [31,33,84]. Based on these findings and our results that CDKN1A overexpression induces G_1_-phase arrest in HPr-1AR and PC3-Lenti-AR cells (S5 and S7 Figs), which are consistent with results from Mirochnik et al. [41], we conclude that AR-mediated inhibition of G_1_/S-phase progression and cell proliferation also involves the CDK inhibitory activity of CDKN1A.

An intriguing difference between HPr-1AR and PC3-Lenti-AR is that the DHT-induced G_1_ arrest of HPr-1AR cells appears to be “transient” in comparison to the G_1_ arrest of PC3-Lenti-AR cells (compare Fig 1G to Fig 7G). The PC3-Lenti-AR data suggests that the DHT-treated PC3-Lenti-AR cells continue through the cell cycle until they arrest in G_1_ phase of the next cycle, which may be due to an absence of p53 in these cells [85,86]. Based on the HPr-1AR cell cycle data, one should not conclude that DHT-induced growth suppression of HPr-1AR is transient. The substantial decrease in phosphorylated RB protein at 24 and 48 hours (Fig 3) together with diminished proliferation of DHT-treated HPr-1AR cell at 2–4 days (Fig 1) provide evidence that AR-mediated growth suppression of HPr-1AR persists for 48 hours or more.

### AR-mediated transcriptional regulation of cyclin D1/2, CDK4/6, and CDKN1A

In a previous study using HPr-1AR, we identified cyclin D1 as an AR-occupied ARG, based on the presence of an AR-occupied binding sequence within 50 kb of its transcription start site (GGAACGtccAGTGCC is 4.8 kb upstream of the transcription start site in intron 3) [43]. This same sequence is also occupied by the closely related glucocorticoid receptor (GR) in A549 cells treated with GR agonist, dexamethasone [87]. GR binds *in vitro* with similar affinity as AR to consensus sequences in the elementary half-sites GGTACAnnnTGTTCT [88]. However, the finding that androgen treatment decreased the half-life of cyclin D1 mRNA (Fig 4) without decreasing cyclin D1 pre-mRNA levels (Fig 5) indicates that post-transcriptional control of mRNA decay [53] is a central mechanism through which AR signaling regulates cyclin D1 expression in HPr-1AR. Cyclin D1 mRNA destabilization and decay could occur by dissociation of mRNA stabilizing factors from cyclin D1 transcripts or by association of an androgen-induced miRNA. The miR-34a miRNA has been shown to target the 3’-untranslated mRNA region of cyclin D1 and decrease its expression [89]. Further, the introduction of miR-34a precursor into PC-3 cells resulted in cell growth inhibition and G_1_-phase arrest [90]. Additional experiments designed to identify sequences in the cyclin D1 mRNA that are responsible for transcript destabilization and decay are needed to define the exact role of AR in this mechanism.

Although the stability of cyclin D2 mRNA was unaffected by DHT treatment, cyclin D2 pre-mRNA was androgen-repressed, implicating AR in the transcriptional repression of cyclin D2 (Fig 5). Although AR occupancy at the cyclin D2 locus was not interrogated in our previous study [43], GR occupancy data from the ENCODE Consortium identify a GR-occupied region 47 kb upstream of the cyclin D2 transcription start site in dexamethasone-treated A549 cells [87]. Indeed, the AR-mediated mechanisms responsible for cyclin D1 and D2 down-regulation by androgen in HPr-1AR differ from the post-transcriptional mechanism that reportedly increases cyclin D1/2 expression in LNCaP prostate cancer cells [91].

We also found that CDK4 and CDK6 nascent transcripts were androgen-repressed in HPr-1AR and PC3-Lenti-AR (Figs 5 and 8), which is consistent with AR-mediated transcriptional repression of these CDK genes. In LNCaP cells, which proliferate with androgen, CDK4 mRNA was previously shown to be androgen-induced [3]. Although we have not interrogated AR occupancy at CDK4 and CDK6 in HPr-1AR and PC3-Lenti-AR, an AR-occupied region was identified 530 bp upstream of the transcription start site of CDK4 in LNCaP cells [92]. The ENCODE Consortium data for dexamethasone-treated A549 cells also identify a GR-occupied region at the transcription start site of the CDK4 gene and 12 or more GR-occupied regions in several introns of the CDK6 gene [87].

CDKN1A expression is indeed androgen-stimulated in HPr-1AR and PC3-Lenti-AR (Figs 3 and 8), which is consistent with previous reports [36,39,91,93]. Transcriptional activation of the CDKN1A gene may involve direct binding of ligand-bound AR at a functional AR binding sequence that was previously identified 200 bp upstream of the transcription start site of transcript variant 1 [63]. In addition to this AR binding sequence being a GR-occupied region in dexamethasone-treated A549 cells, GR occupancy data from the ENCODE Consortium identify 5 additional GR-occupied regions at the CDKN1A locus (3 regions are located 9–33 kb upstream of the transcription start site and 2 regions are in intron 1) [87].

### Conclusions

Multiple mechanisms are responsible for the AR-mediated growth suppression of HPr-1AR and PC3-Lenti-AR. In HPr-1AR, AR-mediated inhibition of proliferation involves down-regulation of cyclin D-CDK complexes through transcriptional repression of cyclin D2, CDK4, and CDK6 mRNAs, destabilization of cyclin D1 mRNA, and transcriptional activation of CDKN1A mRNA. In PC-Lenti-AR, AR-mediated growth suppression occurs through a similar mechanism, albeit without down-regulation of cyclin D1/2 expression. Our findings have revealed a central role for several ARGs in HPr-1AR and PC3-Lenti-AR whereby AR-mediated changes in the expression and activity of cyclin D-CDK4/6 complexes suppress cell cycle progression and proliferation of these prostate epithelial cells. Therefore, future studies should extend our cell culture analysis to assess the AR-mediated regulation of these ARGs and their relative contribution to epithelial cell proliferation in developing and adult prostate tissue, and during neoplastic disease progression in the prostate. It will be important to determine how AR decreases cyclin D1 mRNA stability and represses transcription of the cyclin D2, CDK4, and CDK6 genes and whether these mechanisms can be exploited for therapeutic intervention in prostate neoplasia and malignancy. Future studies designed to address these unknowns will provide additional insight into regulatory mechanisms operating within hormone-responsive gene networks.

## Supporting Information

S1 Fig Regulation of cyclin D, CDK, and CDKN1A mRNAs by androgen in HPr-1AR. After treatment with 10 nM DHT or vehicle control for various durations, total RNA was isolated from HPr-1AR cells, cDNA was synthesized by reverse transcription and the relative levels of cyclin mRNAs were quantified by QPCR analysis. (A) In time course experiments, cyclin D1 and D2 mRNAs were androgen-repressed at 8–48 hours (h). (B) CDK4 and CDK6 mRNAs were down-regulated at 16–48 hours, whereas CDKN1A mRNA was androgen-induced at 24–48 hours. Data represent the mean ± SEM, n = 3. * *P* < 0.05.
(TIF)

S2 Fig Inhibition of HPr-1AR cell proliferation in response to androgen and CDK selective inhibitor. HPr-1AR cells were treated with 10 nM of DHT or vehicle control and various concentrations of CDK selective inhibitor, PD0332991, and the relative number of viable cells was determined after 72 hours of treatment by quantification of ATP in metabolically active cells. Cell number increased for all treatments, however, HPr-1AR proliferation was decreased at 72 hours with increasing concentrations of PD0332991. By itself, PD0332991 inhibited HPr-1AR proliferation at doses ranging from 2–5 μM. However, the combined effects of PD0332991 and DHT on HPr-1AR proliferation were similar to DHT treatment alone. Data represent the mean ± SEM, n = 4. * *P* < 0.05.
(TIF)

S3 Fig AR-mediated destabilization of cyclin D1 mRNA in HPr-1AR. (A) Experimental design scheme depicts transcriptional inhibition by 5,6-dichlororibofuranosylbenzimidazole (DRB), DHT treatment, and mRNA isolation. Cells were treated with transcription inhibitor, DRB, for 1 hour prior to treatment with 10 nM DHT or vehicle control, and total RNA was harvested at the indicated time points for quantification by QPCR. (B) Transcription of the PYGO2 control gene was unchanged by androgen, and the half-life of its mRNAs was unaffected. The half-life of cyclin D2 mRNA was unchanged by DHT treatment compared to vehicle control, whereas the cyclin D1 mRNA half-life was 5.5 hours in DHT-treated samples compared to 11.5 hours in control samples. Data represent the mean ± SEM, n = 3.
(TIF)

S4 Fig Transcriptional regulation of cyclin D1/2 pre-mRNAs by androgen in HPr-1AR. After treatment with 10 nM DHT or vehicle control for various durations, total RNA was isolated from HPr-1AR cells, cDNA was synthesized by reverse transcription and the relative levels of cyclin D1/2 pre-mRNAs were quantified by QPCR analysis. In time course experiments, cyclin D1 pre-mRNA was unaffected by androgen treatment, whereas cyclin D2 pre-mRNAs declined substantially with DHT-treatment. Cyclin D2 pre-mRNA was androgen-repressed to the greatest extent at 24–48 hours (h). Data represent the mean ± SEM, n = 3. * *P* < 0.05.
(TIF)

S5 Fig Overexpression of CDKN1A inhibits cell cycle progression in HPr-1AR. (A) Stable overexpression of CDKN1A was validated by immunoblot analysis. In comparison to parental HPr-1AR cells (black) and RFP control cells (blue), which have endogenous CDKN1A expression, HPr-1AR cells that stably overexpress CDKN1A (orange) have increased (B) forward light scatter and (C) side light scatter values, suggesting that these cells have increased volume relative to the control cells. In comparison to (D) RFP control cells, (E) HPr-1AR cells that stably overexpress CDKN1A have increased DCV DNA intensity, which is consistent with increased DNA content in these cells. In addition, these cells display an abnormal cell cycle profile that interfered with accurate resolution of the cell cycle distribution. The integrated viral vectors used in these experiments also express red fluorescent protein, which allowed for gating and analysis of transduced cells among a background of uninfected cells.
(TIF)

S6 Fig Regulation of CDK and CDKN1A mRNAs by androgen in PC3-Lenti-AR. After treatment with 10 nM DHT or vehicle control for various durations, total RNA was isolated from PC3-Lenti-AR cells, cDNA was synthesized by reverse transcription and the relative levels of CDK mRNAs were quantified by QPCR analysis. In time course experiments, CDK4 and CDK6 mRNAs were significantly androgen-repressed and CDKN1A mRNA was androgen-induced by 6–8 hours. Data represent the mean ± SEM, n = 3. * *P* < 0.05.
(TIF)

S7 Fig Overexpression of CDKN1A inhibits cell cycle progression in PC3-Lenti-AR. (A) Stable overexpression of CDKN1A was validated by immunoblot analysis. In comparison to parental PC3-Lenti-AR cells (black) and RFP control cells (blue), which have endogenous CDKN1A expression, PC3-Lenti-AR cells that stably overexpress CDKN1A (orange) have increased (B) forward light scatter and (C) side light scatter values, suggesting that these cells have increased volume relative to the control cells. In comparison to (D) RFP control cells, (E) PC3-Lenti-AR cells that stably overexpress CDKN1A have increased DCV DNA intensity, which is consistent with increased DNA content in these cells. In addition, these cells display an abnormal cell cycle profile that interfered with accurate resolution of the cell cycle distribution. The integrated viral vectors used in these experiments also express red fluorescent protein, which allowed for gating and analysis of transduced cells among a background of uninfected cells.
(TIF)
