# Supplementary figures and images for: Androgen Receptor-Mediated Growth Suppression of HPr-1AR and PC3-Lenti-AR Prostate Epithelial Cells
Source: PLoS One. 2015 Sep 15;10(9):e0138286. doi: 10.1371/journal.pone.0138286 (PMC4570807; doi:10.1371/journal.pone.0138286)

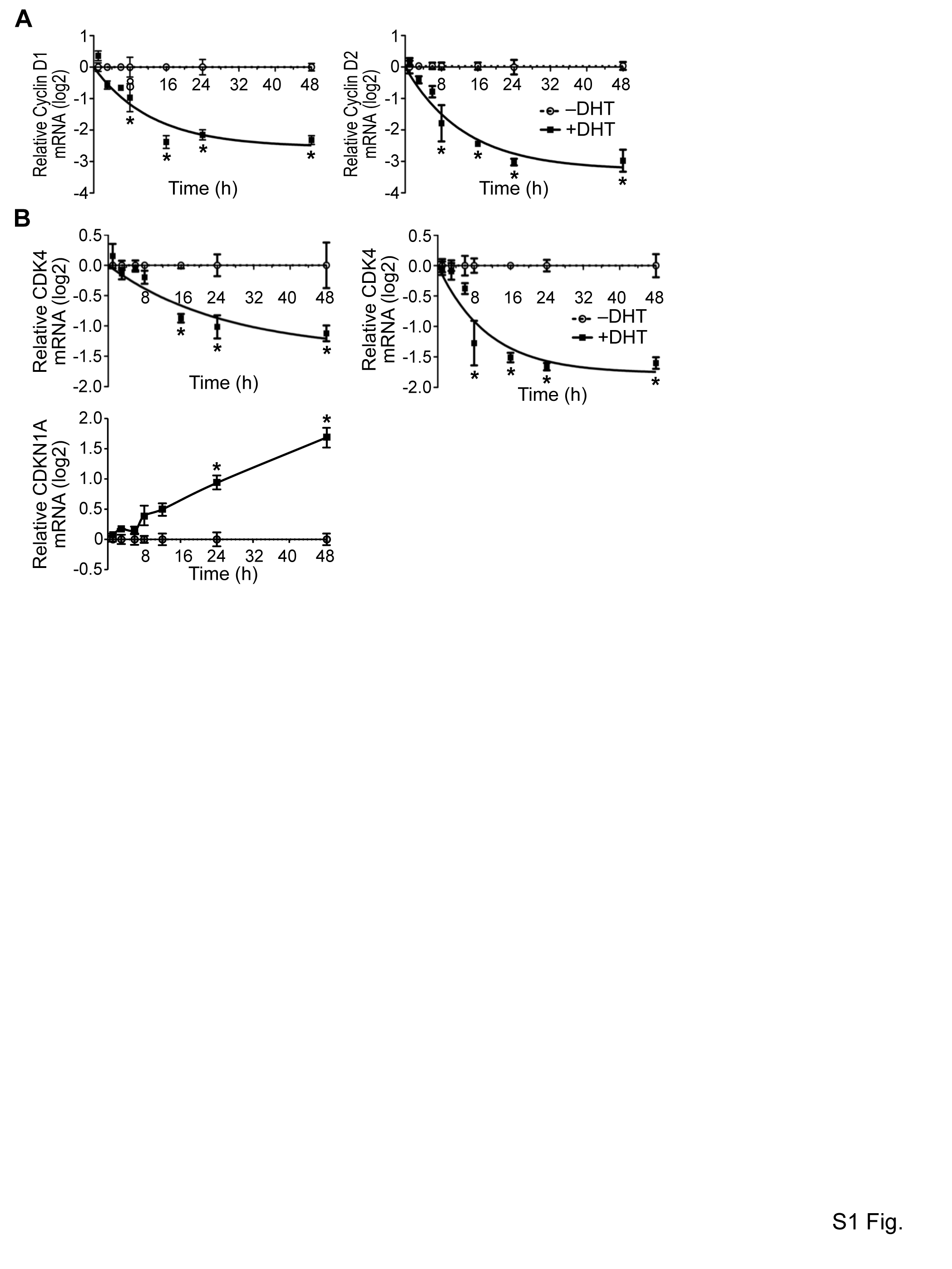

Supplement: S1 Fig — After treatment with 10 nM DHT or vehicle control for various durations, total RNA was isolated from HPr-1AR cells, cDNA was synthesized by reverse transcription and the relative levels of cyclin mRNAs were quantified by QPCR analysis. (A) In time course experiments, cyclin D1 and D2 mRNAs were androgen-repressed at 8–48 hours (h). (B) CDK4 and CDK6 mRNAs were down-regulated at 16–48 hours, whereas CDKN1A mRNA was androgen-induced at 24–48 hours. Data represent the mean ± SEM, n = 3. * P < 0.05. (TIF) [file pone.0138286.s001.tif]

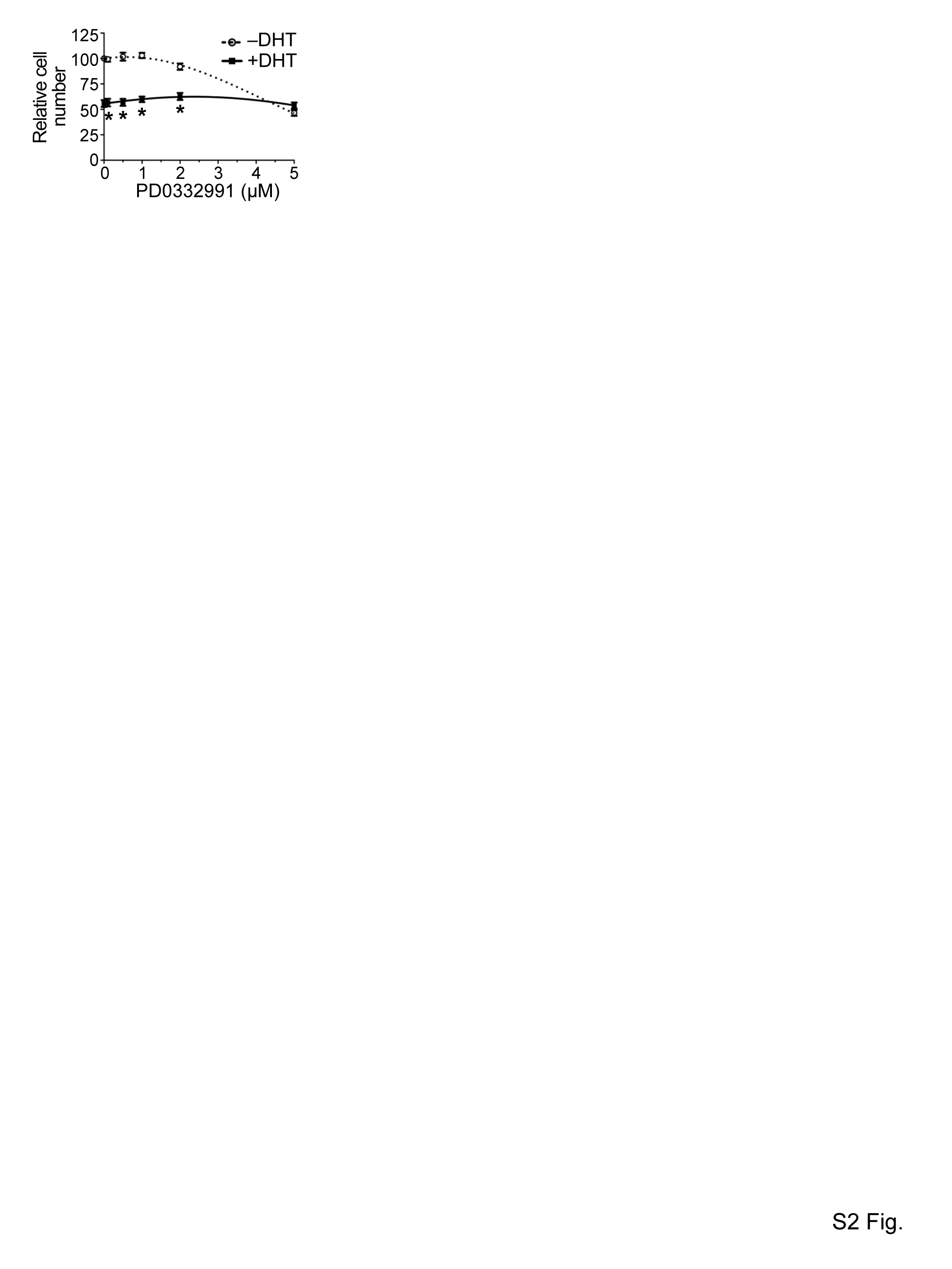

Supplement: S2 Fig — HPr-1AR cells were treated with 10 nM of DHT or vehicle control and various concentrations of CDK selective inhibitor, PD0332991, and the relative number of viable cells was determined after 72 hours of treatment by quantification of ATP in metabolically active cells. Cell number increased for all treatments, however, HPr-1AR proliferation was decreased at 72 hours with increasing concentrations of PD0332991. By itself, PD0332991 inhibited HPr-1AR proliferation at doses ranging from 2–5 μM. However, the combined effects of PD0332991 and DHT on HPr-1AR proliferation were similar to DHT treatment alone. Data represent the mean ± SEM, n = 4. * P < 0.05. (TIF) [file pone.0138286.s002.tif]

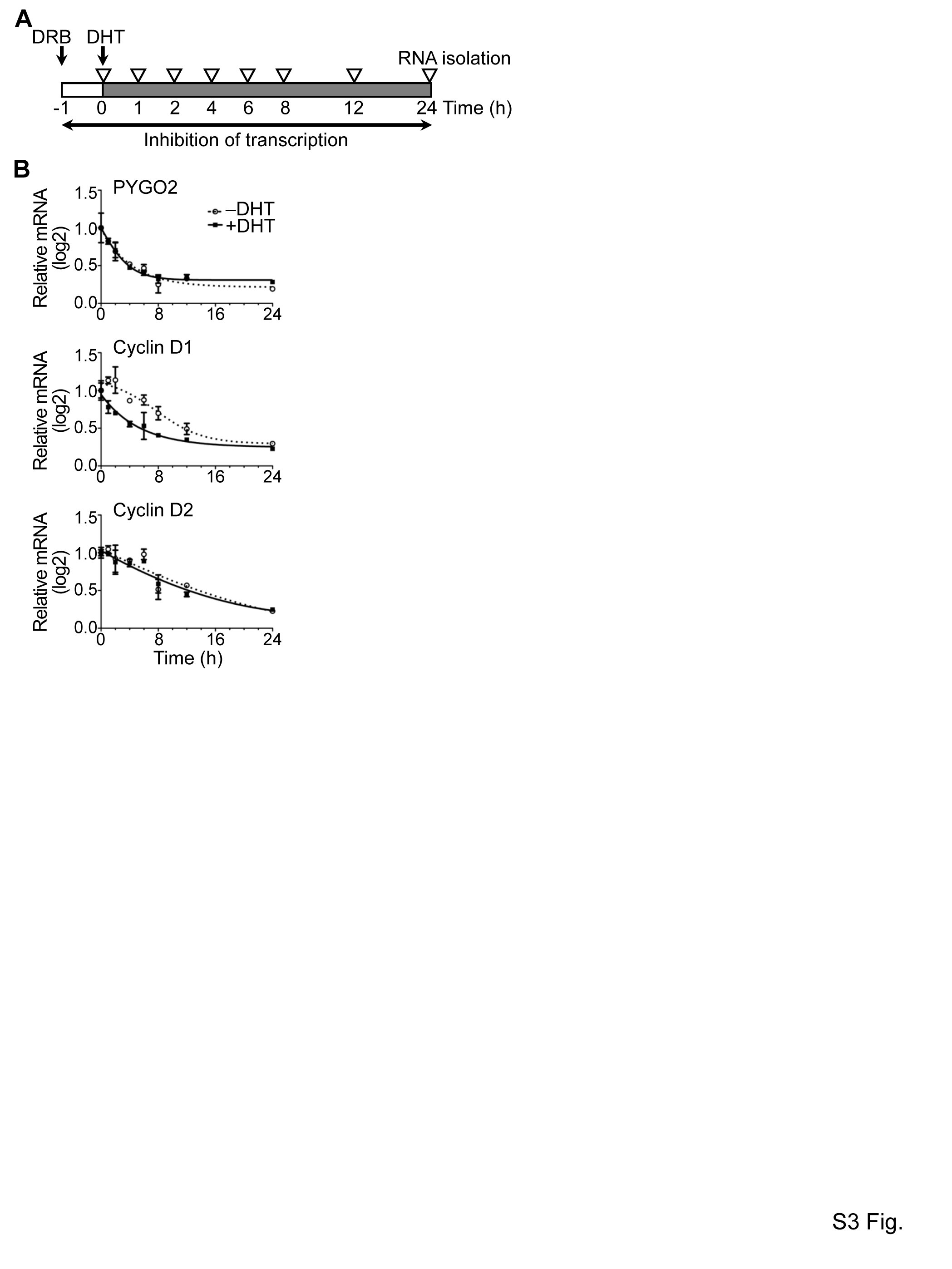

Supplement: S3 Fig — (A) Experimental design scheme depicts transcriptional inhibition by 5,6-dichlororibofuranosylbenzimidazole (DRB), DHT treatment, and mRNA isolation. Cells were treated with transcription inhibitor, DRB, for 1 hour prior to treatment with 10 nM DHT or vehicle control, and total RNA was harvested at the indicated time points for quantification by QPCR. (B) Transcription of the PYGO2 control gene was unchanged by androgen, and the half-life of its mRNAs was unaffected. The half-life of cyclin D2 mRNA was unchanged by DHT treatment compared to vehicle control, whereas the cyclin D1 mRNA half-life was 5.5 hours in DHT-treated samples compared to 11.5 hours in control samples. Data represent the mean ± SEM, n = 3. (TIF) [file pone.0138286.s003.tif]

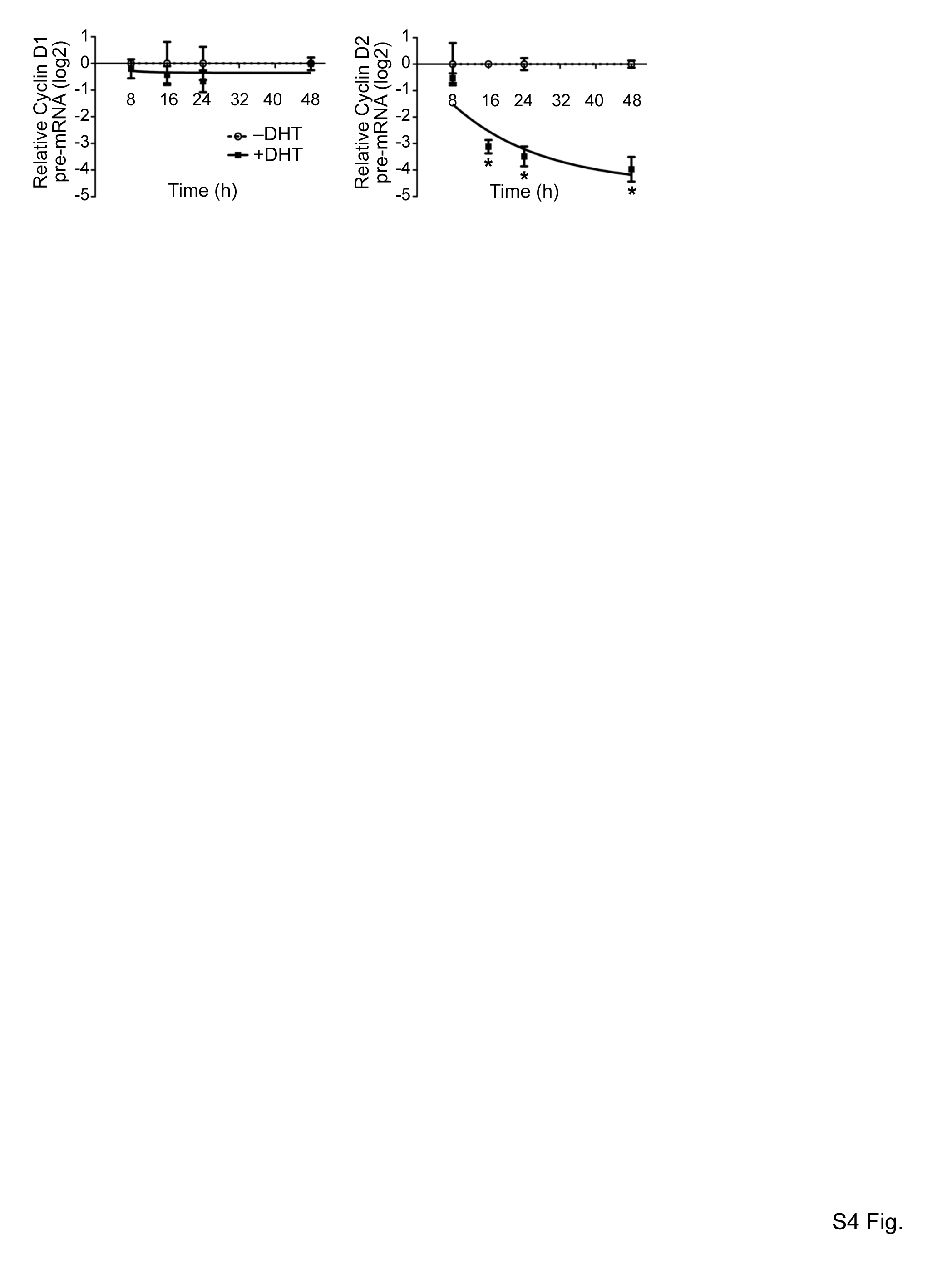

Supplement: S4 Fig — After treatment with 10 nM DHT or vehicle control for various durations, total RNA was isolated from HPr-1AR cells, cDNA was synthesized by reverse transcription and the relative levels of cyclin D1/2 pre-mRNAs were quantified by QPCR analysis. In time course experiments, cyclin D1 pre-mRNA was unaffected by androgen treatment, whereas cyclin D2 pre-mRNAs declined substantially with DHT-treatment. Cyclin D2 pre-mRNA was androgen-repressed to the greatest extent at 24–48 hours (h). Data represent the mean ± SEM, n = 3. * P < 0.05. (TIF) [file pone.0138286.s004.tif]

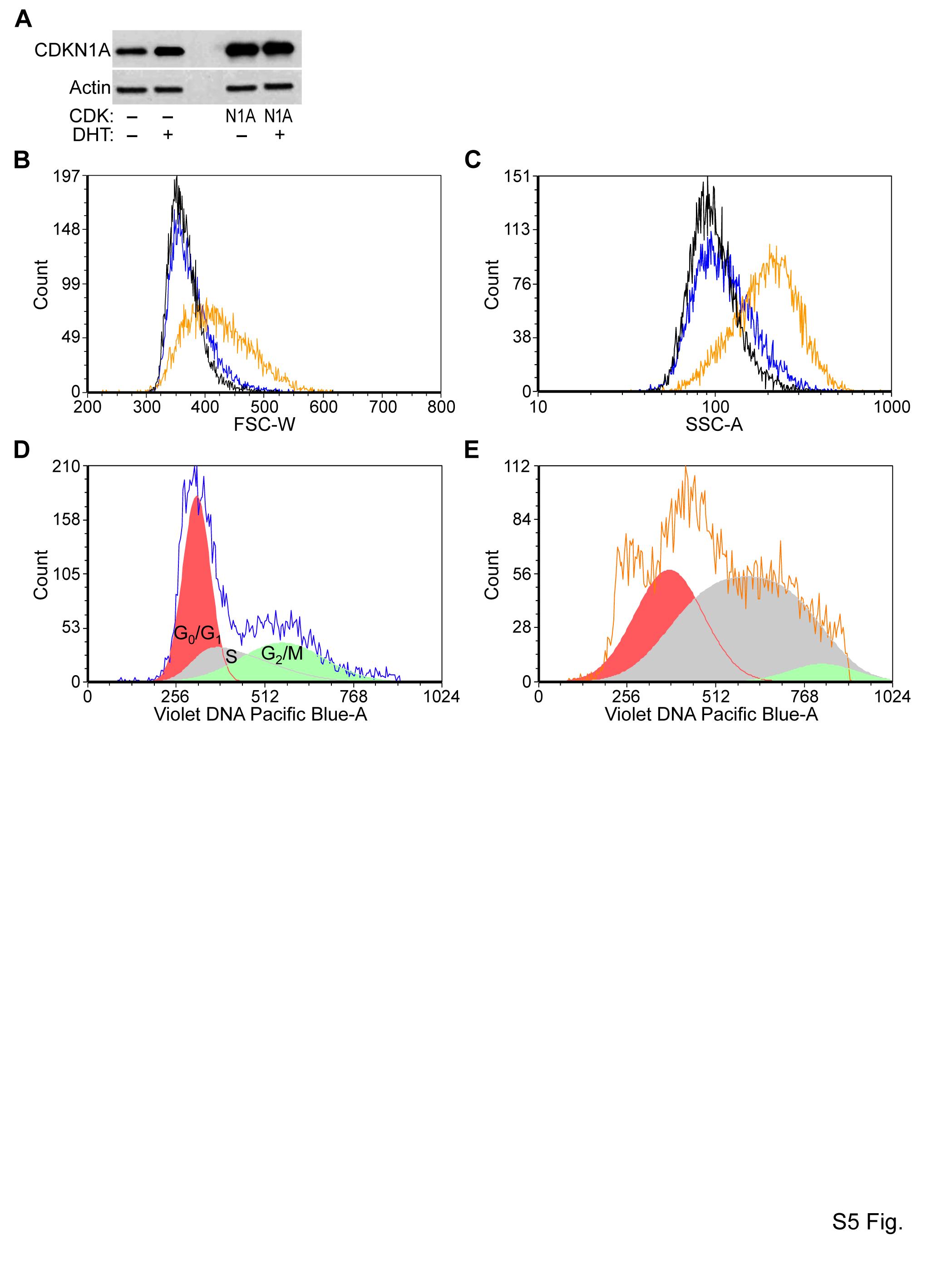

Supplement: S5 Fig — (A) Stable overexpression of CDKN1A was validated by immunoblot analysis. In comparison to parental HPr-1AR cells (black) and RFP control cells (blue), which have endogenous CDKN1A expression, HPr-1AR cells that stably overexpress CDKN1A (orange) have increased (B) forward light scatter and (C) side light scatter values, suggesting that these cells have increased volume relative to the control cells. In comparison to (D) RFP control cells, (E) HPr-1AR cells that stably overexpress CDKN1A have increased DCV DNA intensity, which is consistent with increased DNA content in these cells. In addition, these cells display an abnormal cell cycle profile that interfered with accurate resolution of the cell cycle distribution. The integrated viral vectors used in these experiments also express red fluorescent protein, which allowed for gating and analysis of transduced cells among a background of uninfected cells. (TIF) [file pone.0138286.s005.tif]

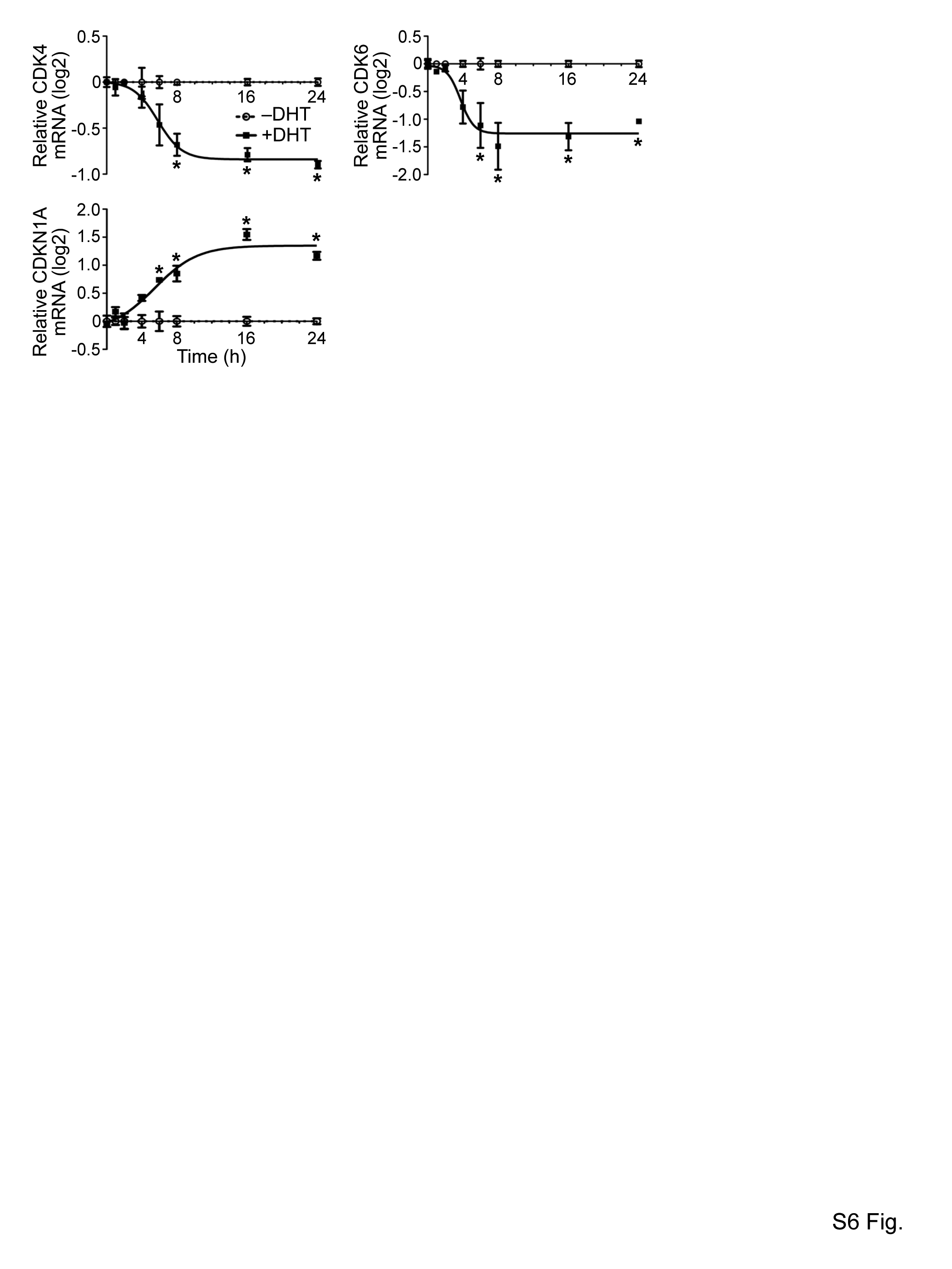

Supplement: S6 Fig — After treatment with 10 nM DHT or vehicle control for various durations, total RNA was isolated from PC3-Lenti-AR cells, cDNA was synthesized by reverse transcription and the relative levels of CDK mRNAs were quantified by QPCR analysis. In time course experiments, CDK4 and CDK6 mRNAs were significantly androgen-repressed and CDKN1A mRNA was androgen-induced by 6–8 hours. Data represent the mean ± SEM, n = 3. * P < 0.05. (TIF) [file pone.0138286.s006.tif]

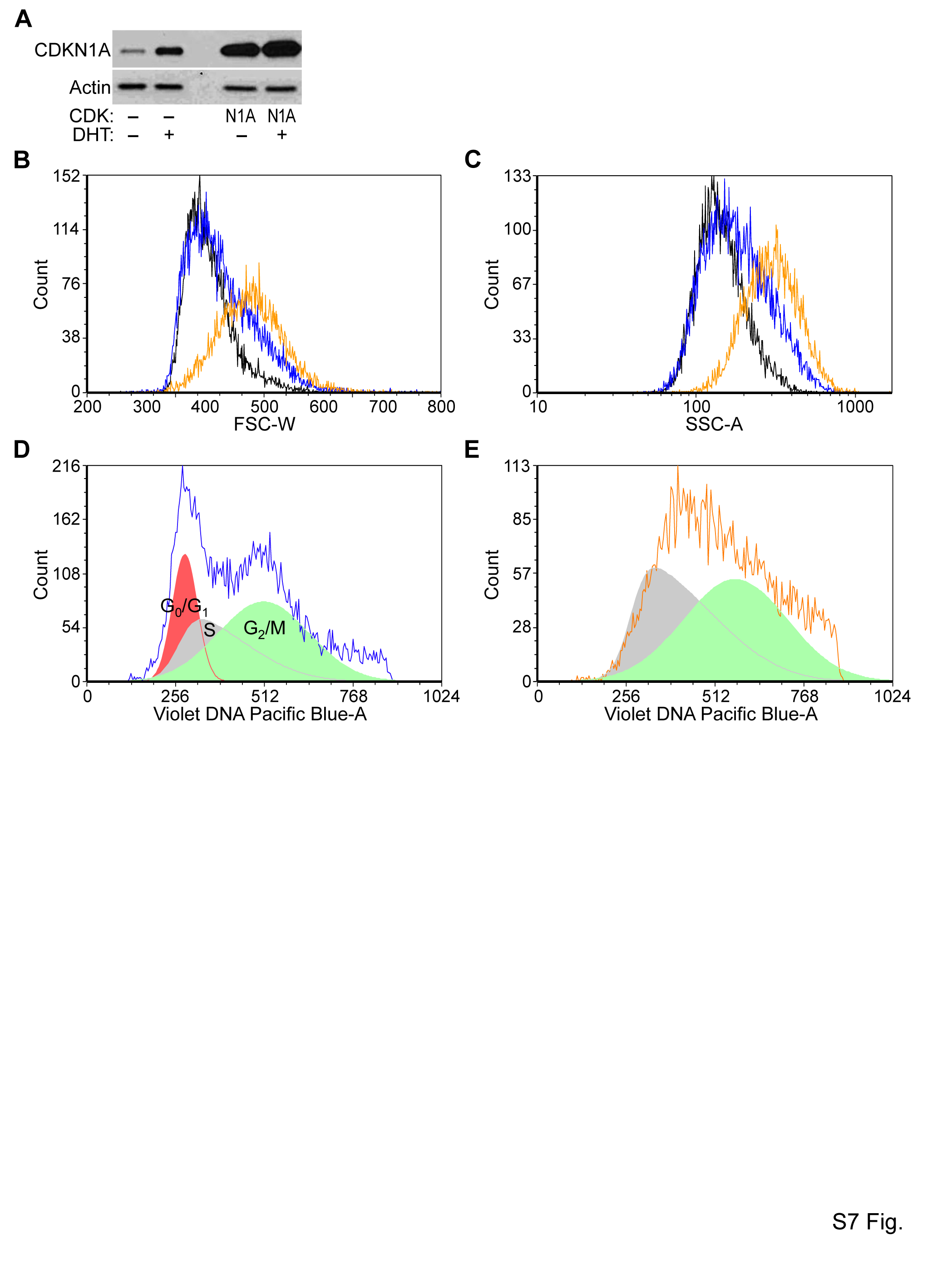

Supplement: S7 Fig — (A) Stable overexpression of CDKN1A was validated by immunoblot analysis. In comparison to parental PC3-Lenti-AR cells (black) and RFP control cells (blue), which have endogenous CDKN1A expression, PC3-Lenti-AR cells that stably overexpress CDKN1A (orange) have increased (B) forward light scatter and (C) side light scatter values, suggesting that these cells have increased volume relative to the control cells. In comparison to (D) RFP control cells, (E) PC3-Lenti-AR cells that stably overexpress CDKN1A have increased DCV DNA intensity, which is consistent with increased DNA content in these cells. In addition, these cells display an abnormal cell cycle profile that interfered with accurate resolution of the cell cycle distribution. The integrated viral vectors used in these experiments also express red fluorescent protein, which allowed for gating and analysis of transduced cells among a background of uninfected cells. (TIF) [file pone.0138286.s007.tif]
